# IL‐33‐Induced TREM2^+^ Macrophages Promote Pathological New Bone Formation Through CREG1‐IGF2R Axis in Ankylosing Spondylitis

**DOI:** 10.1002/advs.202500952

**Published:** 2025-03-17

**Authors:** Wenjun Hao, Siwen Chen, Hua Chao, Zihao Li, Hao Yang, Dongying Chen, Sifang Li, Shuai Zhang, Jingyu Zhang, Jianru Wang, Zemin Li, Xiang Li, Zhongping Zhan, Tangming Guan, Yiwen Zhang, Wende Li, Hui Liu

**Affiliations:** ^1^ Department of Spine Surgery The First Affiliated Hospital Sun Yat‐sen University Guangzhou 510080 China; ^2^ Guangdong Province Key Laboratory of Orthopaedics and Traumatology Guangzhou 510080 China; ^3^ Pediatric Orthopaedics Beijing Jishuitan Hospital Capital Medical University Beijing 102200 China; ^4^ Department of Rheumatology and Immunology The First Affiliated Hospital Sun Yat‐sen University Guangzhou 510080 China; ^5^ Guangdong Laboratory Animals Monitoring Institute Guangdong Key Laboratory of Laboratory Animals Guangzhou 510000 China; ^6^ Institute of Human Virology Department of Pathogen Biology and Biosecurity Key Laboratory of Tropical Disease Control of Ministry of Education Zhongshan School of Medicine Sun Yat‐sen University Guangzhou 510080 China

**Keywords:** ankylosing spondylitis, CREG1, IL33, pathological new bone formation, TREM2^+^ macrophages

## Abstract

Pathological new bone formation is the main cause of disability in ankylosing spondylitis (AS), and so far, it lacks a targeted therapy. Macrophages are central orchestrators of inflammation progression and tissue remodeling, but their contribution to pathological new bone formation has largely not been explored. Here, it is identified that TREM2^+^ macrophages predominated within the sites of new bone formation and adjacent to osteogenic precursor cells. In vivo, both depletion of macrophages and knockout of Trem2 significantly reduced pathological new bone formation in a collagen antibody‐induced arthritis (CAIA) model. Specifically, TREM2^+^ macrophages promoted osteogenic differentiation of ligament‐derived progenitor cells (LDPCs) by secreting CREG1, a secretory glycoprotein involved in cell differentiation and normal physiology. CREG1‐IGF2R‐PI3K‐AKT signaling pathway is involved in TREM2^+^ macrophage‐mediated pathological new bone formation. In addition, it is found that IL‐33 promoted TREM2^+^ macrophage differentiation through phosphorylation of STAT6. Targeting the above signalings alleviated new bone formation in the CAIA model. The findings highlight the critical role of IL‐33‐induced TREM2^+^ macrophages in pathological new bone formation and provide potential therapeutic targets for halting spinal ankylosis in AS.

## Graphical abstract

This study identifies TREM2^+^ macrophages as key contributors to pathological bone formation in ankylosing spondylitis (AS). These macrophages mediate pathological new bone formation via the CREG1‐IGF2R‐PI3K‐AKT pathway and are regulated by IL‐33 through STAT6 phosphorylation. Targeting these pathways reduces pathological new bone formation in the CAIA model, and provides potential therapeutic targets for halting spinal ankylosis in AS.

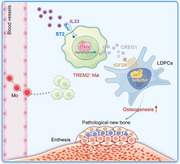

## 1 Introduction

Ankylosing spondylitis (AS) is a chronic inflammatory disease that predominantly affects the axial skeleton and is characterized by inflammatory back pain and spine ankylosis, leading to impaired quality of life and disability.^[^ ^1^, ^2^ ^]^ This disease has affected more than 1.5 million patients and has led to a cost of US$9 billion.^[^ ^3^ ^]^ Pathological new bone formation leading to syndesmophyte growth is the major cause of spinal ankylosis.^[^ ^4^ ^]^ Although nonsteroidal anti‐inflammatory drugs and biologics help to alleviate inflammatory back pain.^[^ ^5^ ^]^ and may halt ankylosis with long‐term administration,^[^ ^6^ ^]^ targeted therapy for pathological new bone formation remains an unmet clinical demand.^[^ ^4^, ^5^, ^7^ ^]^

The relationship between inflammation and pathological new bone is unclear.^[^ ^1^ ^]^ Microtrauma caused by autoimmune responses and mechanical stress is proposed to be the driver of both inflammation and pathological new bone formation.^[^ ^8^ ^]^ Pathologically, new bone formation sometimes decouples from inflammatory responses, as evidenced by the use of anti‐inflammatory treatments, such as tumor necrosis factor inhibitors and interleukin‐17 inhibitors, which halt pathological new bone formation and ankylosis when these agents are administered early and continuously,^[^ ^6^, ^7^ ^]^ suggesting that inflammation and pathological new bone are closely linked.^[^ ^9^ ^]^ Furthermore, our previous study revealed that low‐level inflammation actually promotes pathological new bone formation in AS.^[^ ^10^ ^]^ Significant suppression of pathological new bone formation was observed in animal models whose inflammation was controlled at the initial stage.^[^ ^11^ ^]^ However, the precise mechanism modulating inflammation–osteogenesis crosstalk needs to be further clarified. Understanding the immune cellular and molecular drivers of pathological new bone formation would provide insights into the mechanism of pathological new bone formation in AS and propose a potential therapeutic strategies.

Macrophages, as critical mediators of the immune system, also play key roles in the maintenance of tissue homeostasis and are critical cells involved in the orchestration of chronic inflammation observed in different diseases.^[^ ^12^ ^]^ Macrophages participate in the clearance of debris, antigen presentation, and secretion of multiple cytokines that contribute to tissue regeneration or remodeling in response to niche signals that determine their phenotypes and functions.^[^ ^13^, ^14^ ^]^ Dysregulation of macrophages skews tissue repair to pathological processes such as tissue fibrosis.^[^ ^12^, ^13^ ^]^ As pathological new bone formation intrinsically results in abnormal tissue remodeling,^[^ ^4^ ^]^ macrophages are highly likely involved in this process. Previous studies demonstrated that macrophages expanded in the enthesis fibrocartilage^[^ ^15^ ^]^ and synovium of the sacroiliac joint^[^ ^16^ ^]^ in patients with AS. However, whether a pro‐osteogenic macrophage subset exists in AS and the factors contributing to their differentiation and cellular function are largely unknown.

Here, we identified a subset of TREM2^+^ macrophages that actively contributes to pathological new bone formation through a pro‐osteogenic phenotype. TREM2^+^ macrophages induced new bone formation through the secretion of many previously characterized and uncharacterized pro‐osteogenic proteins. Furthermore, the differentiation of TREM2^+^ macrophages was induced by IL‐33/ST2 signaling. Our findings provide novel macrophage‐related mechanistic insights into pathological new bone formation in AS and a specific player in the connection between inflammation and pathological bone formation, as well as potential therapeutic strategies to prevent or stop the progression of axial skeleton ankylosis.

## 2 Results

### 2.1 Macrophages Accumulate and Play an Essential Role in Pathological Bone Formation

**Figure 1:**
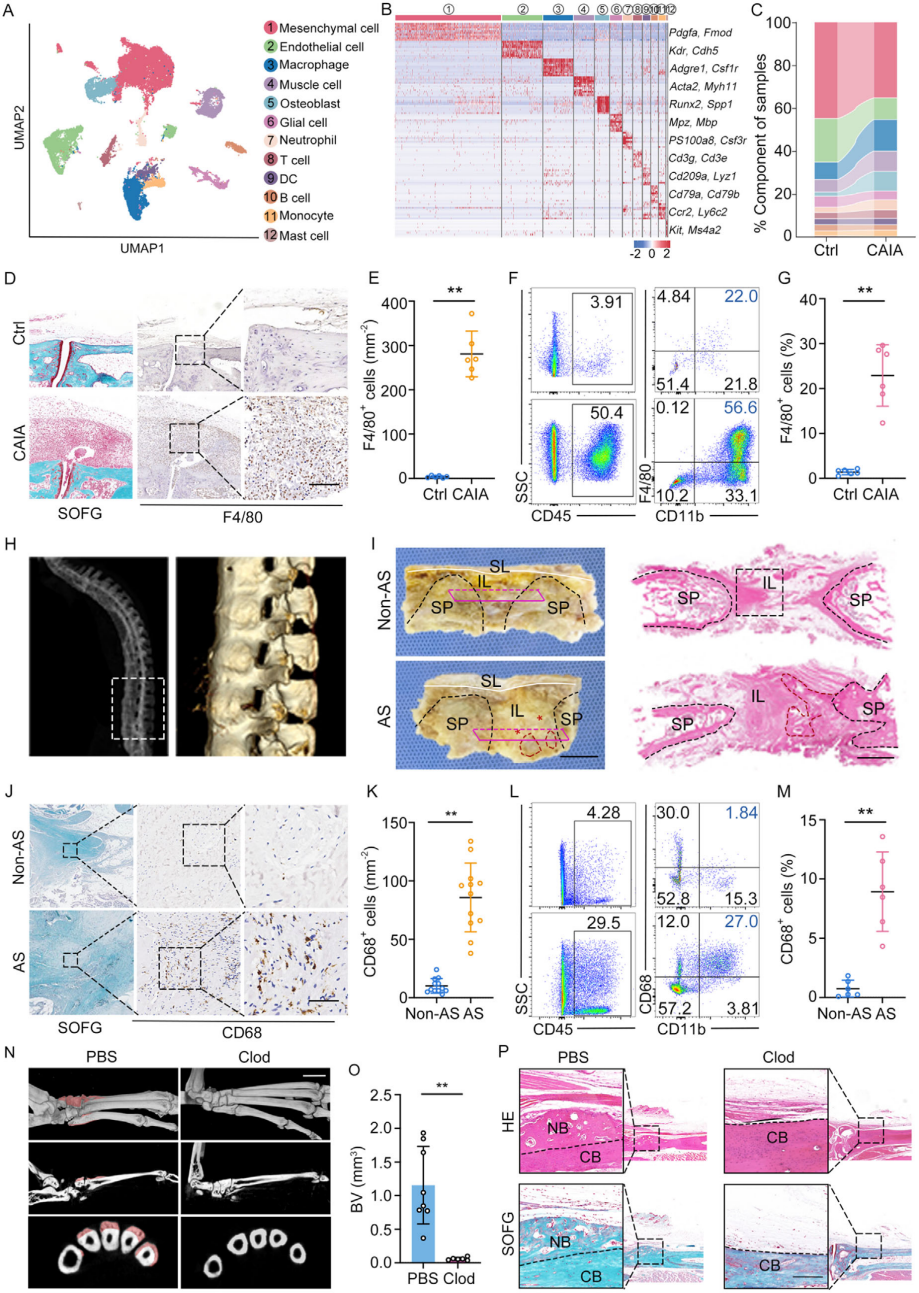
Macrophages are enriched in the enthesis and play an essential role in pathological bone formation. A) Uniform manifold approximation and projection (UMAP) of scRNA‐Seq of all cells from control and CAIA model. Colors and numbers indicate scRNA‐Seq clusters or cell type annotations. B) Heat map of canonical markers of distinct cell clusters. C) Frequencies from scRNA‐seq data of cell subsets from control and CAIA. D,E) SOFG staining, IHC analysis, and quantitative analysis of macrophages in hind paws of CAIA model. n = 6 per group. Scale bar: 100 µm. F,G) Flow cytometry analysis and quantitative analysis of macrophages and in hind paws of CAIA model. n = 6 per group. H) Illustration of human spinal ligament tissue collection. I) Schematic diagram of the anatomy of the spinal ligament and hematoxylin‐eosin (H&E) staining. The red circles indicate pathological new bone; The red asterisks indicate uncalcified ligaments, the potential site of pathological new bone formation; The rose red rectangles represent the location of the H&E on the right. Scale bar: 1.5 cm (left), 5 mm (right). J,K) Safranin O Fast Green (SOFG) staining, IHC analysis, and quantitative analysis of macrophages and in spinal ligament tissues from patients with AS and non‐AS patients. n = 12 per group. Scale bar: 100 µm. L,M) Flow cytometry analysis and quantitative analysis of macrophages and in entheseal tissues from patients with AS and non‐AS patients. n = 6 per group. N,O) µCT images and quantitative analysis of pathological new bone formation in PBS and clodronate‐treated CAIA model. n = 6 per group. Scale bar: 1.5 mm. P) H&E staining and SOFG staining in hind paws of PBS and clodronate‐treated CAIA model. Scale bar: 200 µm. Data is shown as mean±SD. ^**^ *p* < 0.01 determined by unpaired, two‐tailed Student's *t*‐test. AS, ankylosing spondylitis; CAIA, collagen antibody‐induced arthritis; Ctrl, control; IL, interspinous ligament; SL, supraspinous ligament; SP, spinous process; Cold, clodronate; BV, bone volume; CB, cortical bone; NB, new bone.

To elucidate the role of immune cells in this pathological new bone formation, we conducted a comprehensive analysis of single‐cell RNA sequencing (scRNA‐seq) data derived from entheseal tissues of the CAIA model (Figure S1A, supplementary information). Utilizing UMAP (Uniform Manifold Approximation and Projection), gene expression profiles were aligned and visualized in a 2D landscape. Through differential gene expression analysis and leveraging established canonical markers, we manually annotated cell types, encompassing mesenchymal cell, endothelial cell, macrophage, muscle cell, osteoblast, glial, neutrophil, T cell, dendritic cell (DC), B cell, monocyte, and mast cell subsets (**Figure** **1**A,B; Figure S1B,C, Supporting Information). Notably, macrophages emerged as the most abundant immune cell type, exhibiting the most pronounced deviation from the control group (Figure 1C). Immunohistochemical (IHC) staining for F4/80^+^ macrophages and flow cytometry analysis concurred, validating the heightened infiltration of macrophages within the entheses of the CAIA model (Figure 1D–G). To further explore macrophage involvement in AS pathogenesis, we procured spinal non‐calcified ligament tissue from AS patients, a site prone to calcification and pathological bone formation, alongside age‐ and sex‐matched control spinal ligaments obtained during corrective surgeries (Figure 1H,I). Both IHC staining and flow cytometry analysis revealed a marked enrichment of CD68^+^ macrophages in the spinal ligament tissues of AS patients (Figure 1J–M). To assess the contribution of macrophages to pathological new bone formation, we administered liposomal clodronate intravenously in CAIA mice to deplete macrophages. Depletion of macrophages prior or post to arthritis induction significantly mitigated inflammation score and pathological new bone formation in the CAIA model (Figure 1N–P; Figure S2A–G, Supporting Information). These findings underscore the pivotal role of macrophage infiltration in both inflammatory processes and pathological new bone formation.

### 2.2 TREM2^+^ Macrophages are the Predominant Subset During the Process of Pathological New Bone Formation

**Figure 2:**
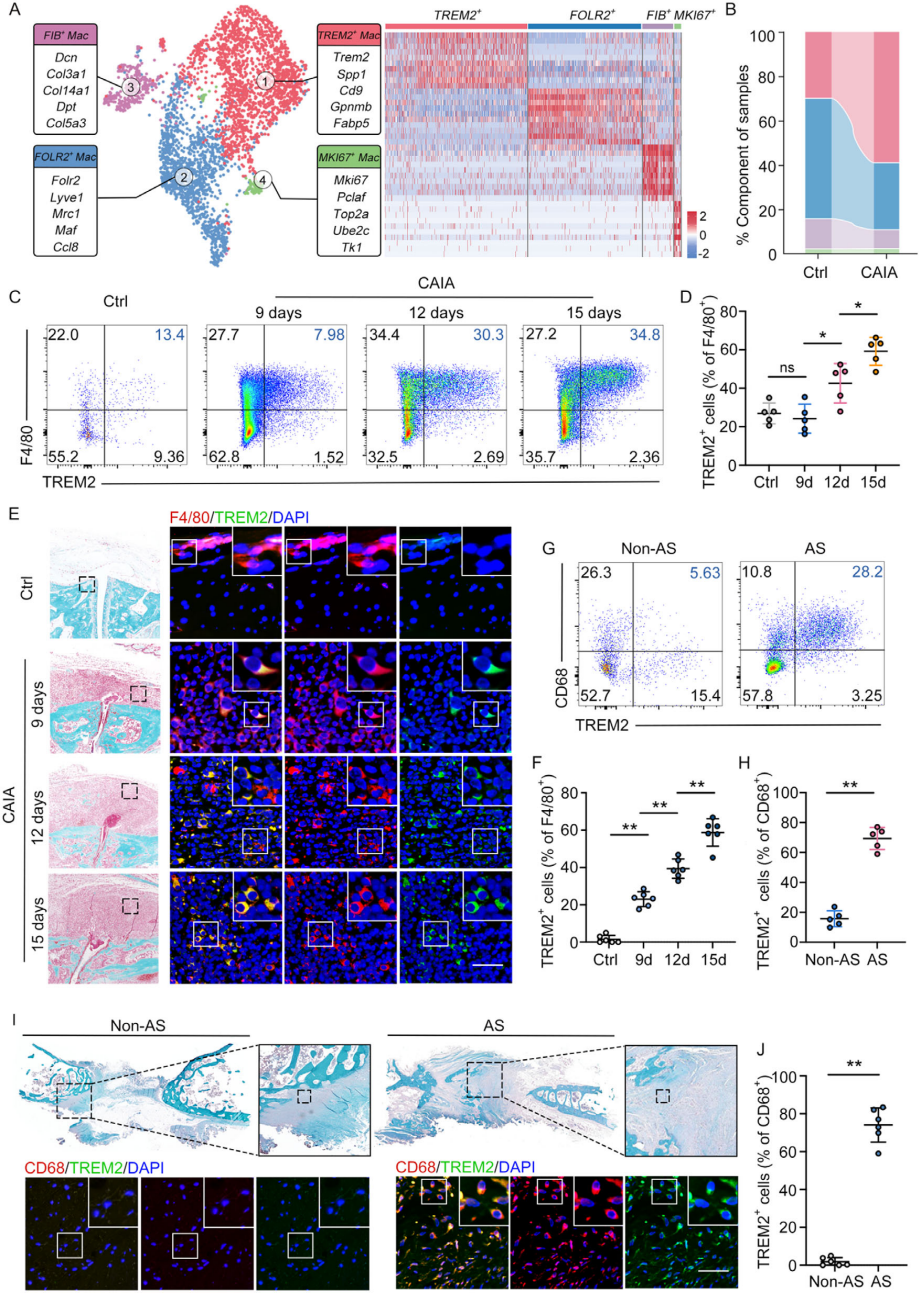
TREM2^+^ macrophages are the predominant macrophage subsets in enthesis from the AS and CAIA models. A) UMAP of scRNA‐seq data from macrophages (left) and heat map of canonical markers of each macrophage cluster (right). B) Frequencies from scRNA‐seq data of macrophage subsets from control and CAIA model. C,D) Flow cytometry analysis and quantitative analysis of TREM2^+^ macrophages in hind paws of control and CAIA model. n = 6 per group. One‐way ANOVA with Tukey's post hoc test. E,F) SOFG staining and double IF staining in hind paws of CAIA model, including staining for F4/80 and TREM2, Scale bar: 100 µm. Semiquantitative analysis of TREM2 colocalization. n = 6 per group. One‐way ANOVA with Tukey's post hoc test. G,H) Flow cytometry analysis and quantitative analysis of TREM2^+^ macrophages in spinal ligament tissues from patients with AS and non‐AS. n = 5 per group. Student's *t*‐test. I,J) SOFG staining and double IF staining in spinal ligament tissues from patients with AS and non‐AS, including staining for CD68 and TREM2, Scale bar: 100 µm. Semiquantitative analysis of TREM2 colocalization. n = 6 per group. Student's *t*‐test. Data is shown as mean±SD. ^*^ *p* < 0.05; ^**^ *p* < 0.01; ns, not significant (P > 0.05). AS, ankylosing spondylitis; CAIA, collagen antibody‐induced arthritis; Ctrl, control.

To further elucidate the roles of macrophage subsets in pathological new bone formation, we re‐clustered macrophages into four distinct subsets. TREM2^+^ macrophages exhibited a gene signature consistent with previously reported markers (SPP1, CD9, GPNMB) and lipid metabolism genes (FABP5, CD36, PLIN2), corresponding to previously described populations promoted tissue repair and remodeling.^[^ ^17^, ^18^, ^19^, ^20^, ^21^, ^22^, ^23^, ^24^ ^]^ FOLR2^+^ macrophages displayed non‐canonical myeloid marker genes (MRC1, LYVE1, CCL8, MAF, F13A1, TXNIP), resembled resident and M2‐like macrophages.^[^ ^23^, ^24^, ^25^ ^]^ They seem to help orchestrating inflammatory and defensing responses. Fib^+^ macrophages, characterized by fibroblast marker genes (DCN, COL3A1, COL14A1), were enriched in genes associated with angiogenesis, collagen biosynthesis, and cell adhesion.^[^ ^26^ ^]^ Mki67^+^ macrophages displayed cell cycle‐related gene expression (TOP2A, MKI67), indicative of proliferative activity (**Figure** **2**A; Figure S3A, Supporting Information).^[^ ^24^ ^]^ Among these subsets, TREM2^+^ macrophages accounted for 58.96% of the total macrophages (Figure 2B). Pseudotime analysis suggested that TREM2^+^ macrophages are derived from monocytes, as seen previously with TREM2^+^ macrophages.^[^ ^11^, ^27^ ^]^ In the CAIA model, immunofluorescence (IF) staining and flow cytometry corroborated the enrichment of TREM2^+^ macrophages at the entheses of CAIA mice. Specifically, flow cytometry data demonstrated a significant increase in the proportion of TREM2^+^ macrophages, from 1.50 ± 2.07% on Day 0 to 58.75 ± 7.35% on Day 15. This augmentation in TREM2^+^ macrophages abundance temporally aligned with the initiation and progression of pathological new bone formation (Figure 2C–F; Figure S3B–D, Supporting Information). Analogously, IF staining and flow cytometry validated the accumulation of TREM2^+^ macrophages in spinal ligament tissues from AS patients (Figure 2G–J). These findings strongly suggest that TREM2^+^ macrophages are the predominant subset driving the process of pathological bone formation.

### 2.3 TREM2^+^ Macrophages Promote Pathological New Bone Formation in a Paracrine Manner

**Figure 3:**
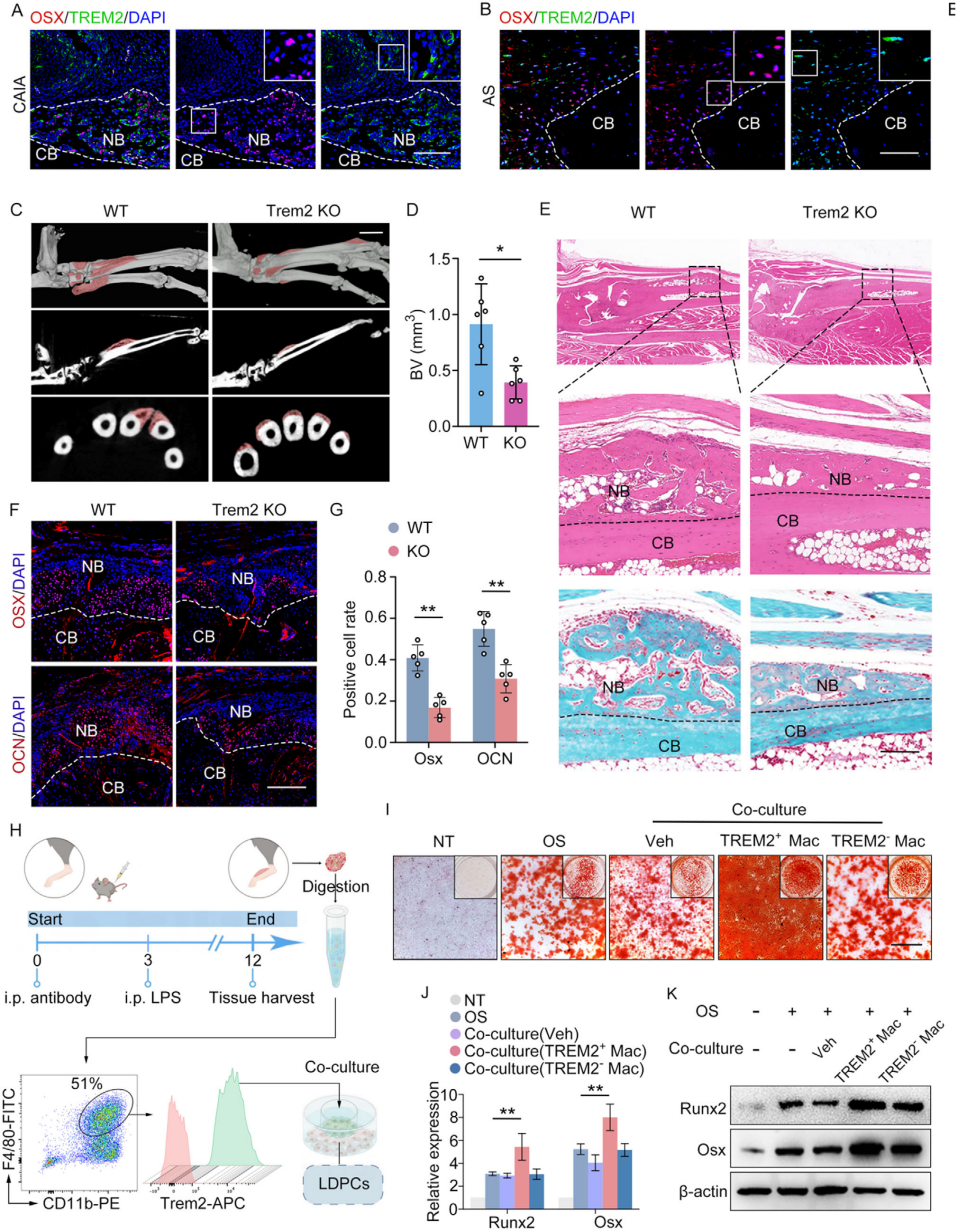
TREM2^+^ macrophages promote pathological new bone formation. A) Double IF staining in hind paws of CAIA model, including staining for TREM2 and Sp7, Scale bar: 200 µm. B) Double IF staining in spinal ligament tissues from patients with AS, including staining for TREM2 and Sp7. Scale bar: 200 µm. C,D) µCT images and quantitative analysis of hind paws of CAIA model with and without Trem2 knockout. n = 6 per group. Scale bar: 1.5 mm. Student's *t*‐test. E) H&E staining and SOFG staining in hind paws of CAIA model with and without Trem2 knockout. Scale bar: 200 µm. F) IF analysis of Runx2, Osx in CAIA model with and without Trem2 knockout. Scale bar: 200 µm. G) Quantitative analysis of Runx2, Osx cell rate. n = 5 per group. Student's *t*‐test. H) Illustration of collection of macrophages and co‐cultured with LDPCs. I) Alizarin Red staining of ligament stem cells co‐cultured with macrophages. Scale bar: 100 µm. J,K) RT‐qPCR analysis and immunoblot analysis of the level of Runx2, Osx of ligament stem cells co‐cultured with macrophages. One‐way ANOVA with Tukey's post hoc test. Data is shown as mean±SD. ^*^ *p* < 0.05; ^**^ *p* < 0.01. AS, ankylosing spondylitis; CAIA, collagen antibody‐induced arthritis; BV, bone volume; Veh, vehicle.

To investigate the role of TREM2^+^ macrophages, we conducted IF staining experiments. We observed significant enrichment and colocalization of TREM2^+^ macrophages and OSX^+^ osteoblasts in the entheseal tissues of the CAIA model and ligament tissue from AS patients (**Figure** **3**A,B). This observation underscores the potential interactions between these two cell types. To elucidate the pivotal role of TREM2^+^ macrophages in pathological new bone formation, we established a CAIA model in Trem2‐knockout (KO) mice. Notably, the volume of pathological new bone was significantly reduced in Trem2‐KO CAIA mice compared to their wild‐type (WT) counterparts (Figure 3C–E; Figure S4A, Supporting Information). Furthermore, the numbers of OSX^+^ or OCN^+^ osteoblasts, were also decreased in Trem2‐KO CAIA mice (Figure 3F,G). To investigate whether TREM2^+^ macrophages promote osteogenesis through a paracrine mechanism, we isolated TREM2^+^ macrophages from the hind paws of CAIA mice using fluorescence‐activated cell sorting (FACS) (Figure S4B, Supporting Information). Meanwhile, we obtained LDPCs in vitro, which possess the capacity for trilineage differentiation (osteogenesis, chondrogenesis, and adipogenesis) (Figure S4C, Supporting Information). We seeded the isolated TREM2^+^ macrophages into the upper chambers of transwell plates and subsequently cocultured them with LDPCs for the purpose of conducting osteogenic differentiation assays (Figure 3H).Alizarin Red staining (ARS) revealed an enhanced mineralization in LDPCs cocultured with TREM2^+^ macrophages. Additionally, reverse transcription‐quantitative polymerase chain reaction (RT‐qPCR) and immunoblot assays demonstrated increased expression levels of osteogenic markers such as Runx2 and Osx in these cocultured LDPCs (Figure 3I–K). Collectively, our data suggested that TREM2^+^ macrophages play a crucial role in promoting pathological new bone formation through paracrine signaling, thereby modulating osteogenic differentiation of LDPCs.

### 2.4 TREM2^+^ Macrophages Promote Pathological New Bone Formation by Secreted CREG1

**Figure 4:**
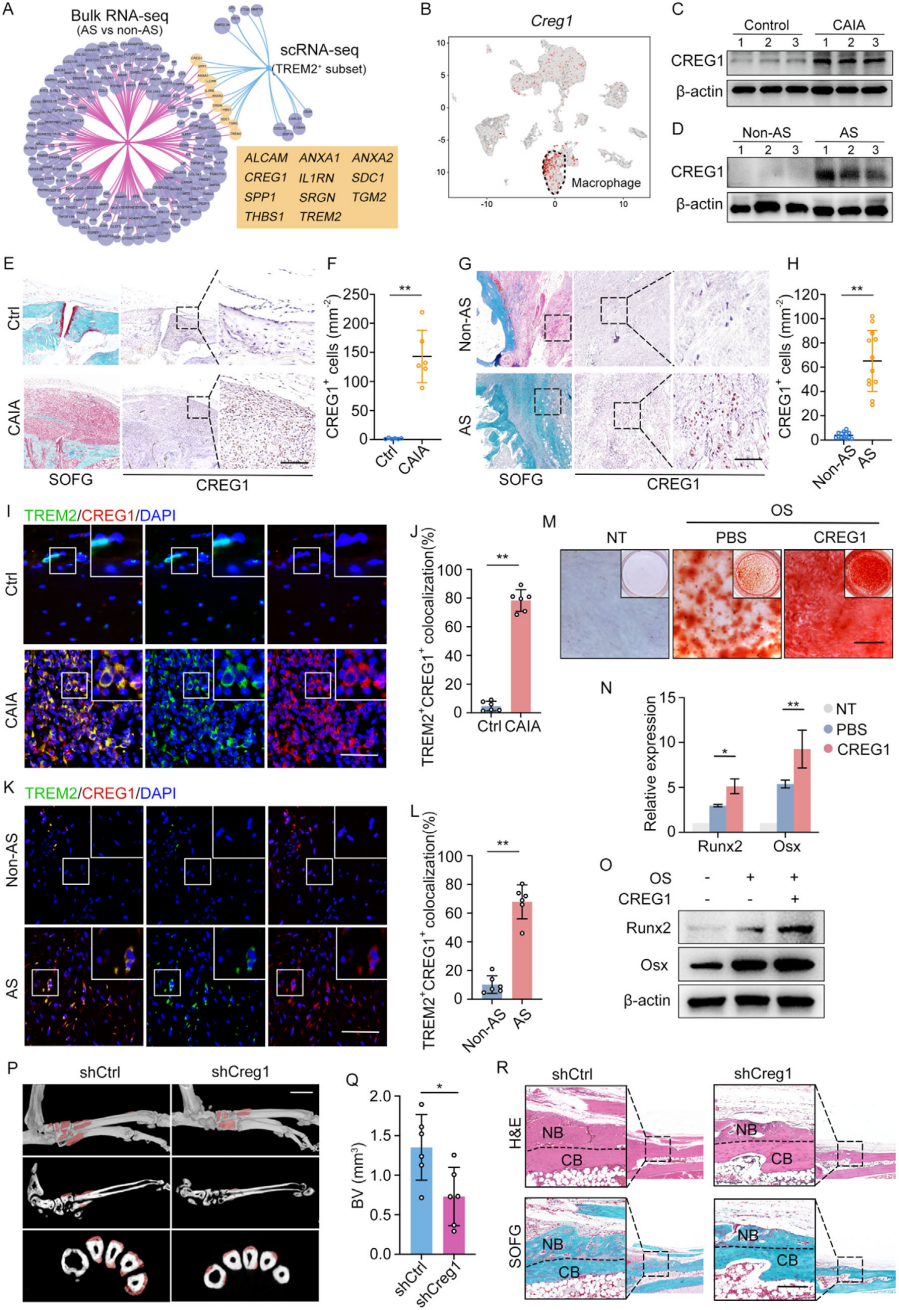
TREM2^+^ macrophages promote pathological new bone formation by secreted CREG1 A) Dynamic network venn diagram of secretory protein upregulated genes form the bulk RNA‐seq of spinal ligament tissue for patients with patients of AS and non‐AS and from the TREM2^+^ macrophage subsets of the scRNA‐seq dataset. B) Feature plots displaying the single‐cell expression of Creg1. C) Immunoblot analysis of expression of CREG1 in hind paws of control and CAIA model D) Immunoblot analysis of expression of CREG1 in spinal ligament tissues from patients with AS and non‐AS patients. E,F) SOFG staining, IHC analysis, and quantitative analysis of CREG1 in hind paws of CAIA model. n = 6 per group. Scale bar: 200 µm. G,H) SOFG staining, IHC analysis, and quantitative analysis of CREG1 in spinal ligament tissues from patients with AS and non‐AS patients. n = 12 per group. Scale bar: 200 µm. I,J) Double IF staining and quantitative analysis in hind paws of CAIA model and control, including staining for TREM2 and CREG1. Scale bar: 200 µm. K,L) Double IF staining and quantitative analysis in spinal ligament tissues from patients with AS and non‐AS, including staining for TREM2 and CREG1. Scale bar: 200 µm. M) Alizarin Red staining of LDPCs treatment with CREG1 for 14 days. Scale bar: 100 µm. N,O) RT‐qPCR analysis and immunoblot analysis of the level of Runx2, Osx of LDPCs treatment with creg1. P,Q) µCT images and quantitative analysis of pathological new bone formation in CAIA model with the administration of shCtrl or shILcreg1 for 4 weeks. n = 6 per group. Scale bar: 1.5 mm. R) H&E staining and SOFG staining in hind paws of shCtrl or shCreg1 treated CAIA model. Scale bar: 200 µm. Data is shown as mean±SD. ^*^ *p* < 0.05; ^**^ *p* < 0.01 determined by unpaired, two‐tailed Student's *t*‐test. AS, ankylosing spondylitis; CAIA, collagen antibody‐induced arthritis; BV, bone volume; CB, cortical bone; NB, new bone.

To further investigate the mechanism underlying TREM2^+^ macrophage‐mediated pathological new bone formation, we employed bulk RNA‐seq to obtain a transcriptional profile of spinal ligament tissue obtained from corrective surgery for patients with AS and age‐matched and sex‐matched controls. This analysis allowed us to pinpoint upregulated genes encoding secreted proteins, not only across the bulk RNA‐seq datasets of AS patients and controls but also within the TREM2^+^ subpopulation identified through single‐cell RNA‐sequencing. By intersecting these secretome profiles from bulk RNA‐seq and TREM2^+^ macrophage subsets in scRNA‐seq dataset, we identified 11 commonly altered secreted proteins (**Figure** **4**A; Table S1, Supporting Information). Notably, among these, CREG1 and TREM2 stood out as primarily macrophage‐derived factors (Figure 4B; Figure S5A, Supporting Information). CREG1 is a secretory glycoprotein that is pivotal for cell differentiation and normal physiological processes. Previous research has illuminated its role in promoting differentiation across various cell types, including embryonic carcinoma cells, vascular smooth muscle cells, and embryonic stem cells. To compare the expression levels of Creg1 between the control group and the CAIA group, we generated violin plots using single‐cell datasets. The results showed that the expression of Creg1 was significantly increased in the CAIA group (Figure S5B, Supporting Information). Additionally, we plotted the violin plots of Creg1 expression across all cell types in both the control and CAIA groups, further confirming that Creg1 was predominantly expressed in macrophages within the CAIA group (Figure S5C, Supporting Information). Creg1 expression was significantly reduced after macrophage elimination (Figure S5D, Supporting Information). Immunoblot assays confirmed the upregulated expression of CREG1 in entheseal tissues of the CAIA model and ligament tissue from AS patients (Figure 4C,D). IHC staining reinforced this finding, highlighting the abundant presence of CREG1 in the CAIA model and AS patients (Figure 4E–H). Further, IF staining of specimens from the CAIA model and AS patients revealed predominant localization of CREG1 expression to TREM2^+^ macrophages (Figure 4I–L). To assess the osteoinductive potential of CREG1, we treated LDPCs with recombinant CREG1 in an osteogenic differentiation culture system. Our results demonstrated that recombinant CREG1 significantly enhanced the osteogenic differentiation of LDPCs, as evidenced by ARS, RT‐qPCR, and immunoblot analyses targeting osteogenic markers Runx2 and Osx (Figure 4M–O). In contrast, TREM2 did not play a significant role in the osteogenic differentiation of LDPCs (Figure S5E, Supporting Information). To validate the functional relevance of CREG1 in vivo, we administered shCreg1 lentivirus via tail vein injection into the CAIA model. IHC staining demonstrated a significant reduction in CREG1 expression after the injection of shCreg1 lentivirus (Figure S5F,G, Supporting Information). This led to a remarkable alleviation of pathological bone formation, as confirmed by µCT, H&E staining, and SOFG staining (Figure 4P–R). These findings emphasize the crucial role of CREG1 in TREM2^+^ macrophage‐mediated pathological bone formation.

### 2.5 CREG1 Promotes Osteogenesis by Activates PI3K‐AKT Signaling

**Figure 5:**
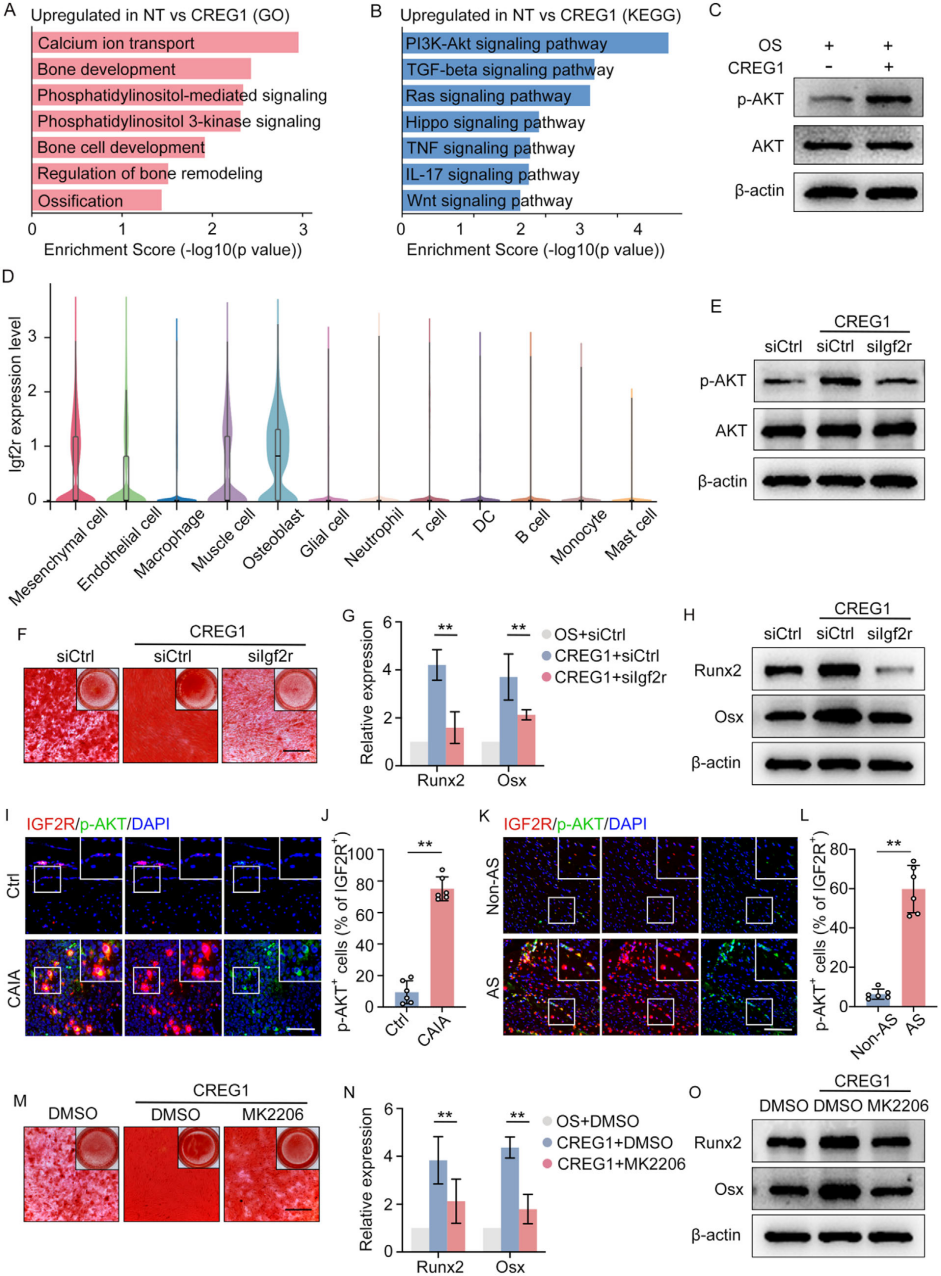
CREG1 promotes osteogenesis by activating PI3K‐AKT signaling. A) GO analysis of upregulated genes in the CREG1 group compared to the PBS group. B) KEGG analysis of upregulated genes in the CREG1 group compared to the PBS group. C) Immunoblot analysis of expression of the level of AKT and pAKT in LDPCs treatment with CREG1. D) Violin plot of Igf2r expression in different clusters in scRNA sequencing. E) Immunoblot analysis of AKT and pAKT level expression after knockdown of IGF2r in LDPCs and treatment with CREG1. F) Alizarin Red staining of LDPCs with knockdown of Igf2r and treatment with CREG1 for 14 days. Scale bar: 100 µm. G,H) RT‐qPCR analysis and immunoblot analysis of the level of Runx2, and Osx of LDPCs with knockdown of Igf2r and treatment with CREG1. I,J) Double IF staining and quantitative analysis in hind paws of CAIA model and control, including staining for IGF2R and pAKT. Scale bar: 200 µm. K,L) Double IF staining and quantitative analysis in spinal ligament tissues from patients with AS and non‐AS, including staining for IGF2R and pAKT. Scale bar: 200 µm. M) Alizarin Red staining of LDPCs with MK2206 and treatment with CREG1 for 14 days. Scale bar: 100 µm. N,O) RT‐qPCR analysis and immunoblot analysis of the level of Runx2, Osx of LDPCs with 抑制剂 and treatment with CREG1. Data is shown as mean±SD. ^*^ *p* < 0.05; ^**^ *p* < 0.01 determined by unpaired, two‐tailed Student's *t*‐test. AS, ankylosing spondylitis; CAIA, collagen antibody‐induced arthritis; Ctrl, control; BV, bone volume; CB, cortical bone; NB, new bone.

To investigate the downstream signaling cascades triggered by CREG1 that promote osteogenesis, we performed Bulk RNA‐seq analysis on LDPCs stimulated with CREG1 under osteogenic induction conditions. Gene Ontology (GO) analysis revealed a significant enrichment of biological processes related to bone development, bone cell differentiation, ossification, as well as phosphatidylinositol‐mediated and phosphatidylinositol 3‐kinase (PI3K) signaling pathways (**Figure** **5**A). Furthermore, KEGG pathway analysis emphasized the enrichment of differentially expressed genes within the PI3K‐Akt signaling pathway, suggesting its potential contribution to the osteogenic effects mediated by CREG1 (Figure 5B). Consistent with these findings, the protein levels of phosphorylated AKT (p‐AKT), a key downstream effector of PI3K, were upregulated upon CREG1 treatment (Figure 5C). Notably, IGF2R (Insulin‐like Growth Factor 2 Receptor) has been established as a receptor for CREG1, and its expression is mainly in mesenchymal cells and osteoblast clusters (Figure 5D). Silencing IGF2R via siIgf2r effectively attenuated the CREG1‐induced phosphorylation of AKT (Figure 5E; Figure S5H,I, Supporting Information), concurrently suppressing the osteogenic differentiation promoted by CREG1 (Figure 5F–H). To further validate the significance of PI3K‐AKT signaling in pathological new bone formation, IF staining for p‐AKT was performed. The results demonstrated an increase in Igf2r^+^ p‐AKT^+^ cells at entheses in both the CAIA model and AS patients (Figure 5I,J). Moreover, inhibiting p‐AKT activity through the application of a specific inhibitor MK2206 mitigated the osteogenic differentiation elicited by CREG1 (Figure 5K–M), reinforcing the central role of PI3K‐AKT signaling in mediating the osteogenic inductive effects of CREG1. In conclusion, these findings highlight the pivotal importance of PI3K‐AKT signaling in facilitating the osteogenic actions of CREG1.

### 2.6 TREM2^+^ Macrophage Differentiation is Induced by IL‐33

**Figure 6:**
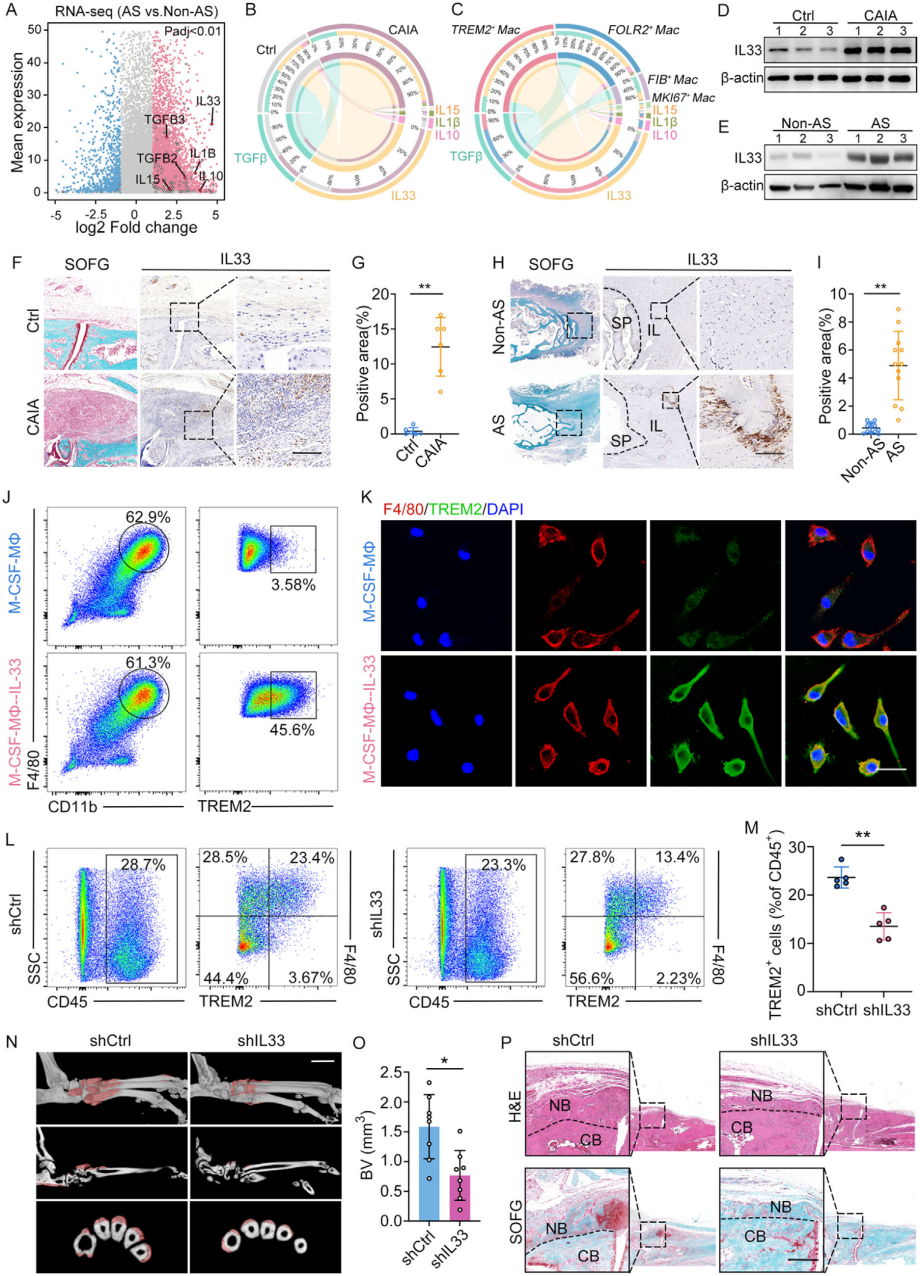
TREM2^+^ macrophage differentiation is induced by IL‐33. A) MA plot of upregulated and downregulated genes from human spinal enthesel tissues of non‐AS and AS groups. Red and blue dots indicate significantly up‐and down‐regulated genes, respectively (adjusted P < 0.01). Red diamonds indicate genes encoding cytokines, black diamonds indicate genes encoding immune‐related. B) Circos plots show the enrichment score of cytokine‐induced gene set in macrophages from both the control and the CAIA model. C) Circos plots show the enrichment score of cytokine‐induced gene set in each subset of macrophages. D) Immunoblot analysis of expression of IL‐33 in human spinal tissue. E) Immunoblot analysis of expression of IL‐33 in spinal ligament tissues from patients with AS and non‐AS patients. F,G) SOFG staining, IHC analysis, and quantitative analysis of IL‐33 in hind paws of CAIA model. n = 6 per group. Scale bar: 200 µm. H,I) SOFG staining, IHC analysis, and quantitative analysis of IL‐33 in spinal ligament tissues from patients with AS and non‐AS patients. n = 12 per group. Scale bar: 200 µm. J) Flow cytometry analysis of TREM2 expression in IL‐33–induced macrophages from BMDMs. K) Immunocytochemical (ICC) staining of BMDMs by TREM2 and F4/80 after IL‐33 treatment for 3 days (shown is one representative result from n = 3 BRs). Scale bar: 20 µm. L) Flow cytometry analysis of TREM2^+^ macrophages in the CAIA model with the administration of shCtrl or shIL‐33 for 10d. M) Quantitative analysis of TREM2^+^ macrophage cells in (J). n = 5 per group. N,O) µCT images and quantitative analysis of pathological new bone formation in CAIA model with the administration of shCtrl or shIL‐33 for 4 weeks. Scale bar: 1.5 mm. n = 8 per group. P) H&E staining and SOFG staining in hind paws of shCtrl or shIL‐33 treated CAIA model. Scale bar: 200 µm. Data is shown as mean±SD. ^*^ *p* < 0.05; ^**^ *p* < 0.01 determined by unpaired, two‐tailed Student's *t*‐test. AS, ankylosing spondylitis; CAIA, collagen antibody‐induced arthritis; Ctrl, control; BV, bone volume; CB, cortical bone; NB, new bone.

The phenotypic characteristics and functional capacities of macrophages are intricately shaped by their microenvironmental cues, such as cytokines, which are exposed during the differentiation process.^[^ ^28^ ^]^ We next investigated cytokines that induce TREM2^+^ macrophage differentiation in the microenvironment. Spinal ligament tissues were obtained from AS patients and controls during surgery for bulk RNA‐seq. Our findings pinpointed TGF‐β, IL‐33, IL‐1, IL‐10, and IL‐15 as the most significantly upregulated cytokines in the AS group (**Figure** **6**A). By projecting the cytokine‐induced gene signatures (Table S2, Supporting Information) of the above cytokines onto the macrophage profile,^[^ ^29^ ^]^ We found that macrophages in the CAIA group exhibited IL‐33‐induced gene signatures (Figure 6B; Figure S6A,B, Supporting Information). These results indicated a crucial role of IL‐33 in the differentiation of macrophages in the CAIA group. Furthermore, TREM2^+^ macrophage subsets demonstrated the highest proportion of cells expressed the IL‐33‐induced gene signature, thereby emphasizing the substantial potential and importance of IL‐33 in driving the differentiation of the TREM2^+^ macrophage subsets (Figure 6C; Figure S6C,D, Supporting Information). To further validate this finding, we confirmed the upregulation of IL‐33 in the entheseal tissues of CAIA model and ligament tissues of AS patients by immunoblot and IHC staining (Figures 6D–I). In vitro studies using murine bone marrow‐derived macrophages (BMDMs) confirmed that IL‐33 induced TREM2^+^ macrophage differentiation, as evidenced by flow cytometry and IF staining assays (Figure 6J,K). The application of ST2 (a classical receptor for IL‐33)‐neutralizing antibodies eliminated IL‐33‐induced TREM2^+^ macrophage differentiation (Figure S6E–G, Supporting Information). Moreover, in vivo knockdown of IL‐33 via tail vein injection of shIL‐33 lentivirus significantly reduced the number of TREM2^+^ macrophages in the CAIA model and mitigated pathological bone formation, as assessed by µCT, H&E, and SOFG staining (Figure 6L–P; Figure S6H,I, Supporting Information).

### 2.7 IL‐33 Induces TREM2^+^ Macrophage Differentiation Through STAT6 Signaling

**Figure 7:**
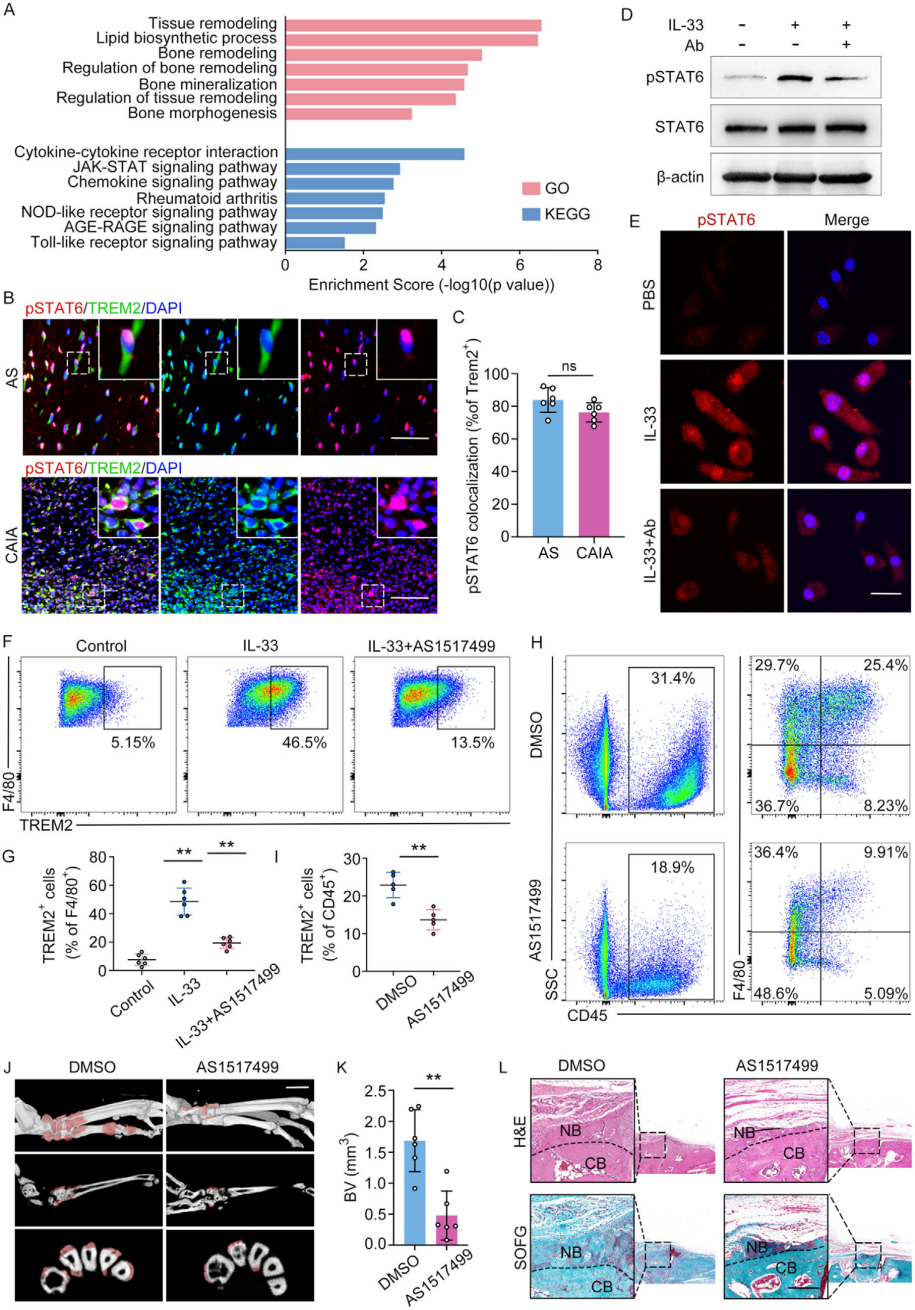
IL‐33 induces macrophage differentiation through STAT6 signaling. A) GO and KEGG analysis of upregulated genes in the IL‐33 group compared to the PBS group. B) Double IF staining of TREM2 and p‐STAT6 in hind paws of the CAIA model and in spinal ligament tissues from patients with AS, n = 6 per group. Scale bar: 3mm. Scale bar: 200 µm. C) Quantitative analysis of TREM2 and p‐STAT6 colocalization. n = 6 per group. D) Immunoblot analysis of STAT6, p‐STAT6 in BMDMs after IL‐33 treatment for 1 h with or without ST2 antibody. E) ICC staining of BMDMs by p‐STAT6 after IL‐33 treatment for 1 h with or without ST2 antibody (shown is one representative result from n = 5 BRs). Scale bar: 20 µm. F) Flow cytometry analysis of TREM2 expression in IL‐33–induced macrophages from BMDMs with the application of AS1517499 or DMSO for 48 h G) Quantitative analysis of TREM2^+^ macrophages in (F). H) Flow cytometry analysis of TREM2^+^ macrophages in the CAIA model with administration of AS1517499 or DMSO for 10 days. I) Quantitative analysis of TREM2^+^ macrophages in (H). J,K) µCT images and quantitative analysis of pathological new bone formation in CAIA model with administration of DMSO or AS1517499 for 4 weeks. n = 6 per group. Scale bar: 1.5 mm. L) H&E staining and SOFG staining in the hind paws of the CAIA model. Scale bar: 200 µm. Data is shown as mean±SD. ^**^ *p* < 0.01; ns, not significant (P > 0.05) determined by unpaired, two‐tailed Student's *t*‐test. AS, ankylosing spondylitis; CAIA, collagen antibody‐induced arthritis; Ctrl, control; Ab, Rat Anti‐Mouse ST2/IL‐33R Monoclonal Antibody; DMSO, dimethyl sulfoxide; AS1517499, STAT6 inhibitor; BV, bone volume; CB, cortical bone; NB, new bone.

To elucidate the mechanisms underlying IL‐33‐mediated TREM2^+^ macrophage differentiation, we performed bulk RNA‐seq on IL‐33‐stimulated BMDMs. GO pathway analyses revealed enrichment in tissue remodeling, bone remodeling, and lipid metabolism were enriched. KEGG pathway analysis revealed enrichment of the JAK‐STAT signaling pathway in the IL‐33‐treated group (**Figure** **7**A). In vivo, TREM2^+^ macrophages were strongly positive for phosphorylation of STAT6 (p‐STAT6) in the CAIA model and AS patients (Figure 7B,C). In addition, IL‐33‐induced STAT6 phosphorylation in BMDMs in vitro, and the application of ST2‐neutralizing antibodies eliminated IL‐33‐induced STAT6 phosphorylation (Figure 7D,E). Inhibition of STAT6 phosphorylation using AS1517499 significantly suppressed IL‐33‐induced TREM2^+^ macrophage differentiation (Figure 7F,G). Since the phosphorylation of STAT6 may be induced by various cytokines, such as IL‐4 and IL‐13, which in turn may be mediated by IL‐33, there is a need to further elucidate the intrinsic connection between the ST2/IL‐33 axis and STAT6 phosphorylation in vivo. We administered shIL33 lentivirus into the CAIA model via tail vein injection. On the 10th day after CAIA induction, the hind paws of the CAIA model were collected, and the levels of IL‐4/IL‐13 were detected by RT‐qPCR and flow cytometry. The results indicated that knockdown IL‐33 in vivo through tail vein injection of shIL‐33 lentivirus significantly reduced the expression levels of IL‐4 and IL‐13 (Figure S7A–F, Supporting Information). Finally, systemic administration of a p‐STAT6 inhibitor to the CAIA model significantly reduced the proportion of TREM2^+^ macrophages (Figure 7H,I) and pathological bone formation (Figure 7J–L), reinforcing the central role of STAT6 activation in IL‐33‐induced TREM2^+^ macrophage differentiation and its consequences on bone remodeling. In conclusion, our findings underscore the dependency of IL‐33/ST2‐mediated TREM2^+^ macrophage differentiation on STAT6 activation.

## 3 Discussion

Pathological new bone formation, a hallmark feature of AS, is an exaggerated response of tissue to mechanical stress and inflammation.^[^ ^1^, ^2^, ^8^ ^]^ Given that inflammatory cytokines, osteogenic molecules, and mechanical stress are associated with pathological new bone formation, inflammation and/or stress‐induced activation of osteogenic signaling pathways are considered to bridge the gap between inflammation and pathological new bone formation.^[^ ^11^, ^30^ ^]^ Nonetheless, the possible role of immune cells has been a long‐standing question.^[^ ^4^, ^8^ ^]^

Macrophages constitute pivotal elements of the innate immune system, renowned for their orchestration of inflammatory responses and tissue remodeling processes.^[^ ^12^ ^]^ Prior investigations have underscored their indispensable function in the progression of inflammation, with Weng et al. demonstrating that the proinflammatory activation of monocyte‐derived macrophages (MDMs) from human blood contributes to exaggerated inflammation in AS.^[^ ^31^ ^]^ Furthermore, Bollow et al. correlated the abundance of macrophages within the synovial region of sacroiliac joint lesions in AS patients with the intensity of MRI‐detected enhancement, suggesting a link to disease activity.^[^ ^16^ ^]^ McGonagle et al. also reported macrophage presence at entheseal sites, where pathological bone formation can potentially initiate.^[^ ^15^, ^32^ ^]^ Nevertheless, the precise pathogenic role of macrophages in the development of pathological new bone in AS remains elusive. Our current study sheds light on this matter by observing macrophage accumulation specifically at sites of pathological new bone formation. Notably, preemptive depletion of macrophages prior to arthritis induction arrested the onset of arthritis, emphasizing their fundamental role in initiating the disease. Moreover, subsequent macrophage depletion post‐arthritis induction significantly impeded new bone formation in an animal model, indicating their active contribution to this pathological cascade. In AS, pathological bone formation is considered as an excessive and abnormal repair, leading to spine ankylosis.^[^ ^4^ ^]^ Macrophages actively participate in this excessive and abnormal repair process, thereby exacerbating the manifestation of an unfavorable disease phenotype.

Considering the heterogeneity of macrophages, we reclustered the macrophages and identified four distinct macrophage subsets: TREM2^+^ macrophages, FOLR2^+^ macrophages, FIB^+^ macrophages, and MKI67^+^ macrophages. Notably, TREM2^+^ macrophages emerged as the predominant subset, characterized by the expression of SPP1, CD9, and GPNMB. This conservative subset has been implicated in various diseases^[^ ^33^, ^34^ ^]^ and originates from circulating monocytes.^[^ ^35^, ^36^ ^]^ TREM2^+^ macrophages exhibit a pivotal role in regulating tissue remodeling, as evidenced by their ability to express matrix metalloproteinases that control fibrotic scar formation^[^ ^20^ ^]^ and regulate collagen remodeling to promote angiogenesis during skin transplantation.^[^ ^22^ ^]^ These findings suggested that TREM2^+^ macrophages may play a crucial part in abnormal tissue remodeling. Since pathological new bone formation intrinsically resulted in abnormal tissue remodeling,^[^ ^4^ ^]^ TREM2^+^ macrophages are likely to participate. TREM2 is a membrane protein that functions in the immune response and is primarily expressed in macrophages.^[^ ^37^ ^]^ In addition, TREM2 is not only a characteristic marker but also an essential driver of the phenotypic acquisition of the TREM2^+^ macrophage subsets. TREM2 deficiency results in markedly reduced TREM2^+^ macrophage gene signatures and abrogated function.^[^ ^27^, ^34^ ^]^ Given that TREM2 is expressed mainly in macrophages, we established a Trem2‐KO mouse model of CAIA to demonstrate the pathological role of this subset. Trem2 KO effectively reduced pathological new bone formation in the CAIA model. Furthermore, our experiments revealed that LDPCs cocultured with TREM2^+^ macrophages exhibited enhanced mineralization, hinting at their pro‐osteogenic potential. This led us to propose that TREM2^+^ macrophages might stimulate LDPCs osteogenesis through a secretory mechanism, further emphasizing their importance in the pathological process of bone formation.

Paracrine regulation of tissue remodeling is important for macrophages.^[^ ^27^ ^]^ TREM2^+^ macrophages express a variety of secretory mediators in different contexts, such as oncostatin M, which maintains hair follicle stem cell quiescence and inhibits hair growth;^[^ ^33^ ^]^ matrix metalloproteinases, which control fibrotic scars in the liver;^[^ ^20^ ^]^ and IL‐18BP, which suppresses NK cell activity, thereby resulting in lung cancer tumor growth.^[^ ^38^ ^]^ Our current findings unravel a novel aspect of the important role for CREG1 in TREM2^+^ macrophages mediated pathologcial new bone formation. In addition, we determined that CREG1 was mainly expressed by TREM2^+^ macrophages. CREG1 is a secretory glycoprotein that is pivotal for cell differentiation and normal physiological processes.^[^ ^39^, ^40^ ^]^ Previous research has illuminated its role in promoting differentiation across various cell types, including embryonic carcinoma cells, vascular smooth muscle cells, and embryonic stem cells, while also positively regulating skeletal muscle regeneration.^[^ ^40^, ^41^, ^42^ ^]^ Additionally, CREG1 is involved in red blood cell development, trophoblast cell proliferation, and differentiation.^[^ ^43^ ^]^ Here, we demonstrate that TREM2^+^ macrophages facilitate pathological new bone formation by secreting CREG1. In both AS patients and the CAIA model, CREG1 is abundantly expressed by TREM2^+^ macrophages and significantly stimulates osteogenic differentiation in LDPCs. Mechanistically, CREG1 exerts its osteogenic induction effect by activating the PI3K‐AKT signaling pathway. Inhibiting either the CREG1 receptor IGF2R or the PI3K‐AKT pathway significantly diminishes the pro‐osteogenic effect of CREG1, highlighting their indispensable roles in TREM2^+^ macrophages mediated pathological bone formation. This underscores the pivotal roles of CREG1 and the PI3K‐AKT signaling pathway in TREM2^+^ macrophages mediated pathological bone formation. Collectively, our data demonstrated that TREM2^+^ macrophages promote pathological new bone formation at the entheseal site by secreting osteoinductive proteins, thereby establishing a microenvironment conducive to osteogenic differentiation. Osteoinductive proteins exhibit distinct mechanisms during different stages of bone formation, such as cartilage hypertrophy, osteogenic differentiation, and mineralization, collectively promoting the occurrence of pathological new bone formation.

Macrophages have diverse phenotypes and functions due to differences in their origin, location, and pathophysiological context.^[^ ^14^ ^]^ Notably, local microenvironmental niches exert a dominant influence on macrophage phenotype. Macrophage phenotype has underscored the pivotal role of local microenvironment signaling in directing the differentiation of TREM2^+^ macrophages. For instance, Do et al. have shown that squalene triggers the differentiation of TREM2^+^ macrophages in acne lesions,^[^ ^23^ ^]^ while Fabre et al. report that GM‐CSF, IL‐17A, and TGF‐β1 collaborate to promote TREM2^+^ macrophages differentiation in organ fibrosi.^[^ ^36^ ^]^ Furthermore, Park et al. discovered that the phagocytosis of apoptotic tumor cells can induce TREM2^+^ macrophage differentiation in lung cancer.^[^ ^38^ ^]^ Here, we identified a strong correlation between upregulated IL‐33 and TREM2^+^ macrophage differentiation. IL‐33, a key player in various inflammatory diseases, is released in response to cellular stress or damage, modulating immune cells to elicit type 2 immune responses crucial for tissue homeostasis and repair.^[^ ^44^, ^45^ ^]^ Here, we found IL‐33 to be abundant in ligament tissues of both the CAIA model and AS patients, with the majority of IL‐33 located extracellularly. Through in vitro experiments, we confirmed that IL‐33 promotes TREM2^+^ macrophage differentiation via its receptor ST2. Inhibiting IL‐33 effectively decreased the proportion of TREM2^+^ macrophages in the CAIA model and attenuated subsequent pathological new bone formation, emphasizing the centrality of IL‐33 in TREM2^+^ macrophage differentiation. Notably, IL‐33 has been targeted in several clinical trials for conditions such as asthma, chronic obstructive pulmonary disease, and atopic dermatitis, which offers potential therapeutic strategies for targeting IL‐33 in the context of pathological new bone formation.

An intricate network of transcription factors, epigenetic alterations, and mRNAs forms the basis for macrophage plasticity and differentiation.^[^ ^46^ ^]^ Previous studies have shown that IL‐33 directly induces STAT6 phosphorylation in vitro.^[^ ^47^, ^48^ ^]^ In our current study, we discovered that STAT6, as a downstream effector of the IL‐33/ST2 pathway, is activated and indispensable for TREM2^+^ macrophage differentiation. Given that the phosphorylation of STAT6 can be induced by various cytokines, including IL‐4 and IL‐13,^[^ ^49^, ^50^ ^]^ which may be mediated by IL‐33,^[^ ^51^, ^52^ ^]^ it is crucial to further investigate the intrinsic link between the ST2/IL‐33 axis and STAT6 phosphorylation in vivo. The results further indicate that knockdown IL‐33 expression in vivo through tail vein injection of lentiviruses carrying shIL‐33 leads to a significant reduction in the expression levels of both IL‐4 and IL‐13. This discovery suggests that IL‐33 not only directly promotes STAT6 phosphorylation but also indirectly promote it in vivo by regulating the IL‐4/IL‐13 pathway. Notably, in vivo, administration of a p‐STAT6 inhibitor (AS1517499) led to a reduction in the proportion of TREM2^+^ macrophages and pathological new bone formation. Therefore, this result emphasizes the role of IL‐33 in inducing the differentiation of TREM2^+^ macrophages through direct or indirect phosphorylation of STAT6.

There are certain limitations to our study. First, as no animal model completely mimics all aspects of the disease, questions may arise regarding the relevance of the model to human spondyloarthritis. Although the CAIA model shares similarities with AS and is commonly used for studying enthesopathy and entheseal pathological new bone formation, the pathological processes and sites of new bone formation are not identical to those in AS patients. However, further investigations are needed to develop an ideal animal model for AS. Second, based on the necessity of the Trem2 gene for phenotyping TREM2^+^ macrophage subsets, we generated a CAIA model in which Trem2 was knocked out to verify the effect of TREM2^+^ macrophage subsets on pathological new bone formation. However, the use of a genetic model of cell‐specific depletion in future studies may provide more definitive evidence to confirm the role of TREM2^+^ macrophages in pathological new bone formation.

In conclusion, we identify a subset of TREM2^+^ macrophages that predominate in the syndesmophyte growth regions of AS patients and promote pathological new bone formation. Elevated IL‐33 promotes TREM2^+^ macrophage differentiation through the ST2/STAT6 axis and results in pro‐osteogenic secretomes. These findings provide novel mechanistic insights into pathological new bone formation and potential therapeutic targets for preventing axial skeleton ankylosis in AS.

## 4 Experimental Section

### Patients

Thirty‐two patients (15 with AS and 17 with non‐AS) were enrolled from the first affiliated hospital of Sun Yat‐Sen University between September 2021 and December 2023. Study procedures were followed in accordance with protocols approved by The Medical Ethics Committee of the First Affiliated Hospital of Sun Yat‐sen University. Assent was obtained from patients. Samples (Peripheral blood, bone, ligamentum flavum, supraspinatus ligament, and interspinous ligament) were collected during surgeries. The indications of surgery for patients with AS included disabling kyphosis, loss of horizontal vision without compensation, painful spinal pseudarthrosis, or Andersson lesion. Non‐AS patients without any systemic inflammatory condition including SpA that fulfilled the indications for correction of scoliosis or spinal decompression of thoracic or lumbar stenosis were obtained.

### Mouse Strains and Model

DBA/1 and C57BL/6J mice were purchased from the Charles River Laboratories. Trem2 knockdown (KO) mice were donated from Professor Nu Zhang's laboratory (Department of Neurosurgery, The First Affiliate Hospital of Sun Yat‐sen University). All animals were housed in standard individually ventilated, pathogen‐free conditions, with 12h: 12 h light cycle, room temperature (21–23 °C), and 40–60% relative humidity. For collagen antibody‐induced arthritis (CAIA) DBA/1 mouse model, DBA/1 mice (male, 6 weeks) were injected intraperitoneally with Arthrogen‐CIA monoclonal antibody cocktail (1.5 mg/20 g) (Chondrex) on day 0, 100 µg lipopolysaccharide (LPS) was injected intraperitoneally on day 3. Control groups were treated with an equivalent volume of non‐specific immunoglobulin (day 0) and LPS (day 3). In certain groups, mice were treated with clodronate (200 µl i.v. every 2–3 days), AS1517499 (10 mg kg^−1^ i.p. daily), shIL‐33 and shCREG1 (100 µl i.v. twice a week). For collagen antibody‐induced arthritis (CAIA) C57BL/6J model, wild‐type (WT) and Trem2‐KO C57BL/6J mice (male, 6 weeks) were injected intraperitoneally with Arthrogen‐CIA monoclonal antibody cocktail (4mg/20 g) (Chondrex) on day 0, 100 µg lipopolysaccharide (LPS) was injected intraperitoneally into each mouse on day 3. At the end of each experimental time point, mice were sacrificed. Hind paw specimens were dissected and fixed with 4% paraformaldehyde for µCT and histological analyses. The Institutional of Ethics Committee (IEC) for Clinical Research and Animal Trials of the First Affiliated Hospital of Sun Yat‐sen University approved all animal experiments.

### Immunohistochemistry

The collected tissues were fixed in 4% PFA for 48 h, specimens were decalcified in 0.5 M EDTA (Sigma–Aldrich) at 4 °C. Paraffin‐embedded tissues were sectioned at 4 µm intervals using a paraffin microtome (RM2235, LECIA). All specimens underwent citrate buffered heat antigen retrieval at 99 °C for 15 min. After washing in PBS, the sections were incubated in 3% hydrogen peroxide for 5 mins for blocking with endogenous peroxidase and followed by washing with PBS. The sections were then incubated against the following antigens: rabbit anti‐human CD68 (1:100, 76437T, CST), rabbit anti‐mouse F4/80 (1:100, GB113373, Servicebio), goat anti‐mouse IL‐33 (1:100, AF3626‐SP, R&D), goat anti‐human IL‐33 (1:100, AF3625‐SP, R&D) overnight at 4 °C. The next day, the sections were washed off with PBS and the slides were probed with Biotinylated secondary antibody and SABC (SA1028, SA1023, BOSTER) reagent for 15 min each at room temperature. Color development was achieved by treatment with the chromogen DAB (AR1027, BOSTER) and was carried out for 3–5 min under a microscope. Hematoxylin staining was also done at the end for nuclear staining. Then, the stained sections were visualized using a digital pathology scanner (KF‐PRO‐020, Kfbio).

### Immunofluorescence

The collected tissues were fixed in 4% PFA for 48 h, specimens were decalcified in 0.5 m EDTA (Sigma–Aldrich) at 4 °C. Paraffin‐embedded or OCT compound‐embedded tissues were sectioned at 4 µm intervals using a paraffin microtome (RM2235, LECIA) or a frozen slicer (HM550VP, MICROM). All specimens underwent citrate buffered heat antigen retrieval at 99 °C for 15 min. After washing in PBS, Blocking was performed with 10% normal donkey serum for 1 h. The tissue sections were incubated overnight at 4 °C in the primary antibodies against following antigens: rat anti‐mouse F4/80 (1:100, ab6640, Abcam), rabbit anti‐human CD68 (1:100, 76437T, CST), goat anti‐mouse TREM2 (1:100, ab95470, Abcam), goat anti‐human TREM2 (1:100, ab85851, Abcam), rabbit anti‐human/mouse CREG1 (1:100, 12220‐1‐AP, Proteintech), p‐stat6 (1:100, ab263947, Abcam) After washing three times in PBS, the primary antibodies were probed with the secondary antibodies Alexa Fluor 594 donkey anti‐Rat (1:100, ab150156, Abcam), Alexa Fluor 488 donkey anti‐Rabbit(1:100, ab150061, Abcam), Alexa Fluor 488 donkey anti‐goat (1:100, bs‐0294D‐AF488, Bioss) and Alexa Fluor 594 donkey anti‐goat (1:100, bs‐0294D‐AF594, Bioss) for 1 h at room temperature. Finally, the coverslips were washed in PBS three times and mounted using Prolong Gold Antifade Reagent containing 4′−6‐diamidino‐2‐phenylindole (Molecular Probes, Invitrogen). Avoid light before being imaged with a confocal microscope (DM6B, LEICA).

### Immunocytochemical

Cells were grown on glass coverslips and fixed for 20 min with 4% PFA (pH 7.4). After washing with PBS, cells were permeabilized using 0.3% Triton X‐100 (Merck Millipore) in PBS for 10 min at room temperature and were blocked with 0.05% Tween 20 (Sigma Aldrich), 1% BSA (Sigma–Aldrich), and 10% donkey serum in PBS for 1 h at room temperature. The cells were incubated overnight at 4 °C in the primary antibodies against the following antigens: rat anti‐mouse F4/80 (1:100, ab6640, Abcam), goat anti‐mouse TREM2 (1:100, ab95470, Abcam), p‐stat6 (1:100, ab263947, Abcam) After washing three times in PBS, the primary antibodies were probed with the secondary antibodies Alexa Fluor 594 donkey anti‐Rat (1:100, ab150156, Abcam), Alexa Fluor 488 donkey anti‐goat (1:100, bs‐0294D‐AF488, Bioss) and Alexa Fluor 488 donkey anti‐Rabbit (1:100, ab150061, Abcam) for 1 h at room temperature. Finally, the coverslips were washed in PBS three times and mounted using Prolong Gold Antifade Reagent containing 4′−6‐diamidino‐2‐phenylindole (Molecular Probes, Invitrogen). Avoid light before being imaged with a confocal microscope (FV3000, Olympus).

### Tissue Processing

Fresh spinal ligament specimens from humans or soft tissue from the dorsal surface of the hind paw from the CAIA mouse model were collected and broken down into smaller fragments. Tissue was then dissociated with 0.2% Type II Collagenase (Worthington) and 0.1% Dispase II (Roche) in Dulbecco's modified eagle medium (DMEM, Gibco) at 37 °C for 2h. Digestions were subsequently quenched with 10% FBS DMEM and filtered through 40µm sterile strainers. Erythrocytes were lysed with ammonium‐chloride‐potassium buffer and single cells were used for flow cytometry.

### FACS and Flow Cytometry

Murine and human cells were washed with phosphate‐buffered saline (PBS) and single cells were then blocked and stained with AF700‐anti‐human CD45 (1:100, 368 513, BioLegend), PE‐anti‐mouse/human CD11b (1:100, 101 207, BioLegend), FITC‐anti‐human CD68 (1:100, 333 805, BioLegend), AF700‐anti‐mouse CD45 (1:100, 368 513, BioLegend), FITC‐anti‐mouse F4/80 (1:100, 123 108, BioLegend), APC‐anti‐mouse/human TREM2 (1:100, FAB17291A, R&D), APC‐anti‐mouse IL‐4 (1:100, 17‐7041‐81, Invitrogen), PE‐anti‐mouse IL‐13 (1:100, 12‐7133‐81, Invitrogen), at room temperature for 30 min, samples were washed and then incubated with zombie violet fixable viability kit (1:500, 102 605, BioLegend) for 10 min at 4°C. Samples were washed and suspended in ice‐cold PBS. Stained cells were run on a FACSAria II (BD) for cell sorting or LSRFortessa for analysis (BD) and analyzed using FlowJo software.

### Cell Treatments

For LDPCs isolation and culture, six‐week‐old C57BL/6 mice were sacrificed, and tail ligament from the tail were mechanically dissected and broken down into smaller fragments. Tissue was then dissociated with 0.2% Type II Collagenase (Worthington) and 0.1% Dispase II (Roche) in Dulbecco's modified eagle medium (DMEM, Gibco) at 37 °C for 2h. filtered through a 40 µm sterile filter and incubated in a complete medium at 37 °C in 5% CO_2_. It was used for subsequent Osteogenic differentiation experiments.

For BMDM isolation and macrophage differentiation, bone marrow cells in femurs from adult mice (7 to 10 weeks old) were collected by flushing with medium (RPMI1640, Gibco) with 10% FBS and filtered through a 40‐µm cell strainer. Incubate in complete medium containing macrophages colony‐stimulating factor‐1 (M‐CSF1) (20 ng ml^−1^, Novoprotein, CB34) at 37 °C in 5% CO_2_. Fresh complete medium was replaced every 3 days and non‐adherent cells were removed. After 7 days, fully differentiated macrophages were obtained. For stimulation experiments, the medium was supplemented with recombinant murine IL‐33 (50 ng ml^−1^, PeproTech, 210‐33), AS1517499 (100 nM ml^−1^, Selleck, S8685). The anti‐ST2‐neutralizing antibodies (2 ug ml^−1^, R&D Systems, MAB10041) were used for blocking experiments.

### Tri‐Lineage Differentiation Assay

To induce osteogenesis, LDPCs were plated in a 24‐well plate at a density of 2 × 10^4^ cells well^−1^ and cultured for 24 h. Subsequently, the original medium was replaced with osteogenic induction medium, which consisted of minimum essential medium supplemented with 10% fetal bovine serum (FBS), 50 µg ml^−1^ L‐ascorbic acid, 0.1 µM dexamethasone, and 10 mM β‐glycerophosphate. This medium was designed to direct the cells toward osteogenic differentiation. Thereafter, fresh osteogenic induction medium was exchanged every 3 days. After 14 days, the cells were stained with alizarin red to observe and verify the osteogenic differentiation.

To induce chondrogenesis, an in vitro chondrogenesis technique was employed, which involves seeding 4 × 10^5^ LDPCs in 50ul droplets using a micromass culture system and placing them in the center of a 6‐well plate. The cells adhere to the plate for 1 to 3 h at 37 °C. Following this, a chondrogenic differentiation medium (AAPR219‐500, Pythonbio) was added to the wells. To maintain optimal cell growth conditions, the medium was changed every 3 days. After 10 days of culture, the micromasses were stained with alcian blue to assess the chondrogenic differentiation.

To induce adipogenic differentiation, LDPCs were plated in a 24‐well plate at a density of 2 × 10^4^ cells well^−1^ and continuously cultured until the cells reached 100% confluence. Subsequently, the medium was replaced with adipogenic induction medium A (HUXMX‐90031, Cyagen), which was used for 3 days of induction. After that, medium A was switched to medium B for 1 day. Then, medium B was switched back to medium A, and this alternating use of A and B was continued to promote adipogenic differentiation. After 21 days, the cells were stained with oil red O to observe and confirm the adipogenic differentiation.

### Osteogenic Differentiation and Alizarin Red Stain

LDPCs were plated in a 24‐well plate at a density of 2 × 10^4^ cells well^−1^ and cultured for 24 h. The cells were then switched to an osteogenic medium consisting of α‐minimum essential medium supplemented with 10% FBS, 50 µg ml^−1^ L‐ascorbic acid, 0.1 µm dexamethasone, and 10 mm β‐glycerophosphate to induce osteogenesis. To evaluate the pro‐osteogenic function of CREG1, adding the corresponding recombinant proteins: mouse CREG1 (100 ng ml^−1^, CM14, NovoProtein), human CREG1 (100 ng ml^−1^, CB02, NovoProtein), human TREM2 (100ng ml^−1^, C807, NovoProtein) to the osteogenic differentiation culture system. The medium was changed every 3–4 days. To detect the mineralization, cells were washed three times with PBS and fixed with 70% ethanol for 10 min. After three washes with distilled water, the cells were stained with alizarin red S (Sigma‐Aldrich) solution (pH 4.1) for 10 min to visualize matrix calcium deposition. The remaining dye was washed three times with distilled water, and the stained cells were photographed.

### In Vitro Macrophages/Ligament Derived Progenitor Cells Co‐culture

A total of 2 × 10^4^ LDPCs were seeded into 24‐well plates and cultured for 24 h. The cells were then switched to 800ul osteogenic medium.

Fresh soft tissue from the dorsal surface of the hind paw from the CAIA mouse was collected 12 days after modeling and broken down into smaller fragments. Tissue was then dissociated with 0.2% Type II Collagenase (Worthington) and 0.1% Dispase II (Roche) in Dulbecco's modified eagle medium (DMEM) medium (Gibco) at 37 °C for 2h. Digestions were subsequently quenched with 10% FBS DMEM and filtered through 40µm sterile strainers. Erythrocytes were lysed with ammonium‐chloride‐potassium buffer and single cells were used for FACS. The obtained macrophages 8 × 10^4^ per well into 0.4‐µm pore inserts of 24‐well trans‐well plates (Corning, 3413) in 200 µl of mixture medium (1:1 RPMI 1640 medium and osteogenic medium). The medium was changed every three days. After 2 weeks, LDPCs were stained with Alizarin Red. In parallel samples, LDPCs from each group were collected at 24 h and 3 days for RT‐qPCR and protein analysis.

### RNA Purification, RNA‐Seq, and Real‐Time qPCR

Tissue RNA extraction was performed with a Tissue RNA Purification Kit Plus (EZBioscience, EZB‐RN001‐plus) according to the manufacturer's instructions, and cell RNA extraction was performed with an Express RNA Purification Kit (EZBioscience, B0004DP). RNA quality was assessed with NanoDrop ND‐1000 and samples with sufficient integrity were used for library construction with the KAPA Stranded RNA‐Seq Library Prep Kit (Illumina). The library was tested and quantified, and reads were aligned using STAR aligner with the mm10 mouse genome. Transcript assembly and differential expression were determined using DESeq2. For reverse transcription (RT) – qPCR, 1ug RNA was reverse‐transcribed used for reverse transcription using a Color Reverse Transcriptase Kit (EZBioscience, A0010CGQ). cDNAs were mixed with the indicated primers and SYBR Green qPCR Mix (EZBioscience, A0012‐R2), and RT‐qPCR was performed on a CFX 96 touch Real‐time PCR System (BIO‐RAD). cDNAs were normalized to equal amounts using primers against Gapdh, Runx2, Osx or Il1rl1. The following primer sequences were used (5′‐3′): Gapdh forward: AGGTCGGTGTGAACGGATTTG, Gapdh reverse: TGTAGACCATGTAGTTGAGGTCA; Runx2 forward: AACGATCTGAGATTTGTGGGC, Runx2 reverse: CCTGCGTGGGATTTCTTGGTT; Osx forward: ATGGCGTCCTCTCTGCTTG, Osx reverse: TGAAAGGTCAGCGTATGGCTT; Il1rl1 forward: TGACACCTTACAAAACCCGGA, Il1rl1 reverse: AGGTCTCTCCCATAAATGCACA; IL‐4 forward: CGTCTGTAGGGCTTCCAAGG, IL‐4 reverse: AGGCATCGAAAAGCCCGAA; IL‐13 forward: TTGCATGGCCTCTGTAACCG, IL‐13 reverse:TGGCGAAACAGTTGCTTTGTG;

### shRNA Sequences for Gene Knockdown

For gene knockdown experiments, DNA oligos of the following target sequences were synthesized (5′‐3′): the scramble shRNA: CAACAAGATGAAGAGCACCAA; shIl33: CCATAAGAAAGGAGACTAGTT; shCreg1: CGCTGGCCACTATCTCCACAATAAA.

### siRNA Knockdown of Igf2r

Small interfering RNA (siRNA) duplexes were predesigned and constructed by MedChemExpress (MedChemExpress, HY‐RS06622). The siRNA would be verified to be efficient before all experiments. Cells were plated in 6‐well plates at a concentration of 1 × 10^5^ cells well^−1^ and transduced with the siRNA using INTERFERin siRNA transfection reagent (Polyplus‐transfection, 101 000 028) following the manufacturer's instructions. Varying amounts of 20 µM siRNA duplexes were mixed with 12 µl well^−1^ of transfection reagent and Opti‐MEM reduced serum medium (Invitrogen, USA) to a total volume of 200 µl, then incubated for 15 min. The mixture was applied to the cells for 24 h at 37 °C in 5% CO_2_.

### Protein Extraction and Immunoblotting

Tissues were homogenized and proteins were extracted using RIPA lysis buffer (Servicebio, G2002) with protease inhibitors (Servicebio, G2006) and phosphatase inhibitors (Servicebio, G2007), followed by centrifugation. Supernatants were quantified using a BCA Protein Assay Kit (Beyotime Biotechnology, P0010). Proteins were separated on a 12% SDS‐PAGE gel and transferred to PVDF membranes. Membranes were blocked and incubated with primary antibodies including mouse‐anti‐runx2 (1:1000, ab76956, Abcam); and mouse‐anti‐osx (1:1000, ab209484, Abcam). goat‐anti‐human IL‐33 (1:1000, AF3625‐SP, R&D), rabbit‐anti‐P‐stat6 (1:1000, ab263947, Abcam), rabbit‐anti‐STAT6(1:1000, ab32520, Abcam), overnight at 4 °C, then with HRP‐conjugated secondary antibodies for 1 h at room temperature. Immunolabeling was detected using an ECL reagent (Meilunbio, MA0186).

### Single‐Cell RNA‐seq Analysis—Preprocessing, mapping, and initial QC

Tissues harvested from entheseal tissues from the CAIA model were chopped it with a blade. The chopped pieces were transferred to a 50 ml centrifuge tube, and preheated collagenase type II and IV (Collagenase II sigma C6885‐500MG, Collagenase IV sigma C5138‐500MG) at 2 mg ml^−1^, CaCl_2_ solution at 100mg ml^−1^ plus 15ul, and DNase I (sigma DN25‐100MG) at 1mg ml^−1^ were added. The mixture was well mixed and placed on a shaker at 37 degrees for 25–40 min. Digestions were subsequently quenched with 10% FBS DMEM and filtered through 40 µm sterile strainers. The mixture was centrifuged at 400 g for 4 min, and the pellet was resuspended with 5 ml RBC Lysis buffer. After lysing on ice for 7 min, it was centrifuged at 300 g for 5 min. The cells were then washed in PBS with 0.04% BSA, counted, and resuspended at a concentration of ≈1000 cells µl^−1^. Cell viability was assessed using Trypan blue exclusion, and only samples with >85% viability were processed for further sequencing.

scRNA‐seq libraries were generated using a microfluidics‐based approach on the Chromium Single‐Cell Controller (10X Genomics) with the Chromium Single Cell 3′ Reagent Kit v3.1, following the manufacturer's instructions. Single cells were encapsulated in gel beads in emulsion (GEMs) and lysed, followed by RNA barcoding, reverse transcription, and PCR amplification. The concentration of the scRNA‐seq libraries was determined using Qubit 3.0, and the distribution of library product fragments was assessed using the Agilent 2100 High Sensitivity DNA Assay Kit (Agilent Technologies, CA, USA). The qualified library was sequenced on an Illumina HiSeq platform.

De‐multiplexing, alignment to the mm10 mouse transcriptome, and UMI‐collapsing were performed using the Cellranger toolkit (version 3.1.0, 10X Genomics). Cells for downstream processing and filtered out low‐quality cells and cell doublets were selected that had 1) a low number (<1000) of unique detected genes, and 2) a high mitochondrial content (10%) determined by the ratio of reads mapping to the mitochondria. Expression values were then internally normalized to 10000 transcripts to create TPM‐like values and finally calculate log2(TPM+1) values.

### PCA, Clustering, UMAP, and Differential Gene Analysis

Merge, normalize data, and scale data were used in the Seurat package to integrate the two datasets for analysis. A graph‐based clustering approach was employed, leveraging PCs to calculate cell distances and embedding cells in an SNN graph based on similar gene expression. The SNN graph was then partitioned into clusters using the Louvain method, refined with Jaccard distance, and visualized via UMAP. Significantly upregulated genes were identified in each cluster using the Wilcoxon rank sum test, requiring at least 1.28‐fold overexpression, expression in >25% of target cluster cells, and a p‐value < 0.05.

### GO Enrichment Analysis

GO (Gene Ontology) was an international standardized gene functional classification system that provides a controlled vocabulary and defined concepts to describe gene and gene product properties across organisms. It consists of three ontologies: molecular function, cellular component, and biological process. GO enrichment analysis identifies significantly enriched GO terms in differentially expressed genes compared to the genome background, using hypergeometric testing and FDR correction (FDR ≤ 0.05 as the threshold). This analysis helps recognize the main biological functions of differentially expressed genes.

### KEGG Enrichment Analysis

KEGG enrichment analysis was conducted on differential genes, selected based on a criterion of log2FC > 1 and p < 0.05. Following this, all peak‐associated gene was systematically mapped to the comprehensive KEGG (Kyoto Encyclopedia of Genes and Genomes) database, and KEGG enrichment analysis was performed on this gene set. The calculated p‐value was FDR corrected with FDR ≤ 0.05 as the threshold.

### Screening for Secreted Proteins

Utilizing the Human Protein Atlas database (accessible at https://www.proteinatlas.org/), a meticulous analysis was conducted to identify upregulated genes encoding secreted proteins within the transcriptome sequencing data derived from AS patients, comparing them with controls. In parallel, single‐cell RNA sequencing (scRNA‐seq) datasets were delved to delineate subset‐specific genes that encode secreted proteins specifically within the TREM2^+^ macrophage subsets. To pinpoint common secreted proteins, an intersection analysis was subsequently performed between these secretome profiles obtained from bulk RNA sequencing and the TREM2^+^ macrophage subsets.

### Generation of Gene Signatures from the Literature

Cytokine signatures were defined as genes that were significantly induced by cytokine treatment. For the IL‐33 activation signature, induced genes were identified between IL‐33 treated and control eosinophils (Bouffi et al., 2013) (GSE43660), as genes were significantly different under an FDR‐corrected t‐test (*P* < 0.01) and that had a log2(fold‐change in expression) >1 (IL‐33 over control).

TGF‐β activation signature was defined previously (Butovsky et al., 2014). For IL1 activation signature, microarray profiles of human monocyte‐derived macrophages treated with IL1 were analyzed (GSE8515). Significantly induced genes were defined as genes with a mean fold‐change ≥1.5 (cytokine over control) and P < 0.05.

For IL10 and IL15 activation signatures, microarray profiles of human monocytes treated with IL10 or IL15 for 6 h (Montoya et al., 2014) (GSE59184) were analyzed. Significantly induced genes were defined as genes with a mean fold change ≥3 (cytokine over control) and P < 0.01.

### AUCell

AUCell, to score the activity of gene sets on single cells. AUCell was a specifically designed analytical tool for scRNA‐seq data, which evaluates whether a particular input gene set was significantly enriched among the expressed genes in each cell by calculating the “Area Under the Curve” (AUC). The AUCell algorithm was utilized to assign a gene set score to each cell. By computing the AUC value for a specific gene set in each cell, AUCell assesses whether these gene sets were significantly enriched in the cell's expressed genes. A higher AUC value indicates more active expression of the gene set within the cell. Based on the AUCell scoring results, gene sets specifically active in different cell types or states were identified. The expression patterns of various gene sets across different cell subpopulations were visualized using Circos plots.

### µCT and Histologic Analyses

All specimens were obtained and subsequently fixed with 4% paraformaldehyde. For µCT scanning, specimens were fitted in a cylindrical sample holder and scanned using a Scanco µCT40 scanner set to 55 kVp and 70 µA. The segmented data were reconstructed as 3D images using MicroCT Ray V3.0 software (Scanco Medical). The quantitative analysis involved drawing a volume of interest and calculating bone volume after automatically determining the threshold. For histologic analysis, specimens were decalcified in 0.5 M EDTA (Sigma–Aldrich) at 4 °C. Paraffin‐embedded sections were stained with haematoxylin and eosin (H&E) and Safranin O Fast Green (SOFG) to evaluate general structures and bone formation. IHC analysis of the specimens was conducted using specific antibodies.

### Statistical Analysis

All data obtained from experiments repeated at least three times was represented as mean±SD. Differences between the two groups were analyzed using a two‐tailed Student's *t*‐test. One‐way analysis of variance with Tukey's post hoc test was used to compare differences between multiple groups. Inflammation scores were analyzed as repeated measurements with generalized estimating equations. The level of significance was set at p < 0.05. All graphs were generated using Prism V.7 (GraphPad), and all statistical tests were performed using SPSS V.27 (IBM).

## Conflict of Interest

The authors declare no conflict of interest.

## Author Contributions

W.H., S.C., and H.C. contributed equally to this work. H.L. conceived the ideas for experimental designs and is responsible for the overall content as the guarantor and developed the concept, supervised the project, and conducted data analysis. W.H., S.C., and H.C. conducted the majority of the experiments, analyzed data, and prepared the manuscript. W.H., S.C., Z.L., and S.Z. conducted sample collection and performed statistical analysis. H.Y., D.C., J.W., Z.L., X.L., Z.Z., T.G., Y.Z., and W.L. provided critical suggestions and instructions for the project and helped compose the manuscript. W.H., J.Z., and S.L. provided µCT analysis. W.H. and S.C. conducted the most animal experiments and performed analysis.

## Supporting information



Supporting Information

## Data Availability

The data that support the findings of this study are available from the corresponding author upon reasonable request.
