# Supplementary material for: IL‐33‐Induced TREM2+ Macrophages Promote Pathological New Bone Formation Through CREG1‐IGF2R Axis in Ankylosing Spondylitis
Source: Adv Sci (Weinh). 2025 Mar 17;12(18):2500952. doi: 10.1002/advs.202500952 (PMC12079337; doi:10.1002/advs.202500952)
Supplement: Supplementary file 1 — Supporting Information [file ADVS-12-2500952-s001.docx]

**Supplementary Materials**

**
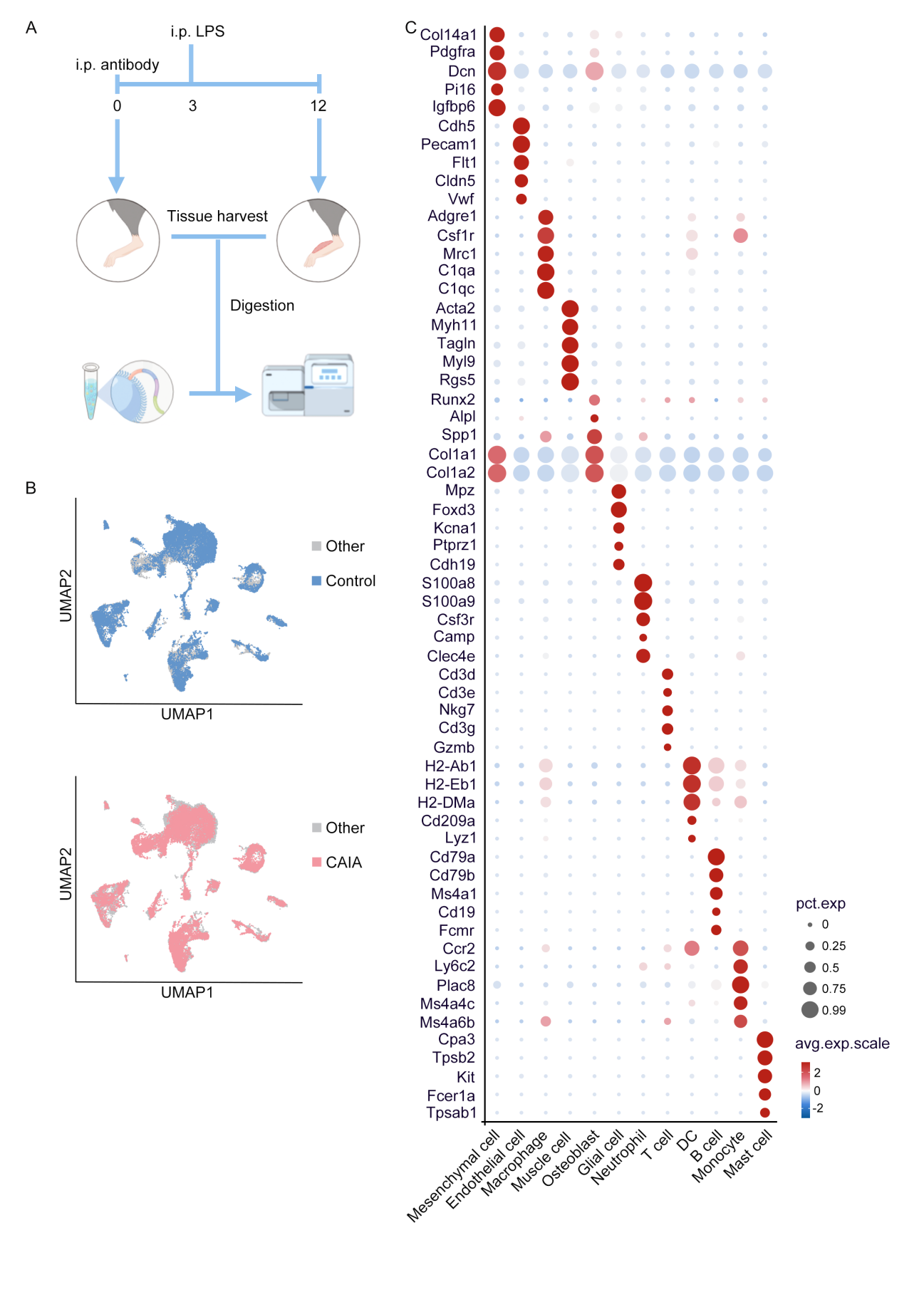
**

**Figure S1.** (**A**) Schematic diagram for scRNA-seq of CAIA model. (**B**) UMAP plot of cells from control and CAIA. control cells are shown in blue, whereas CAIA cells are shown in red. (**C**) Bubble diagram of key markers of each cluster.


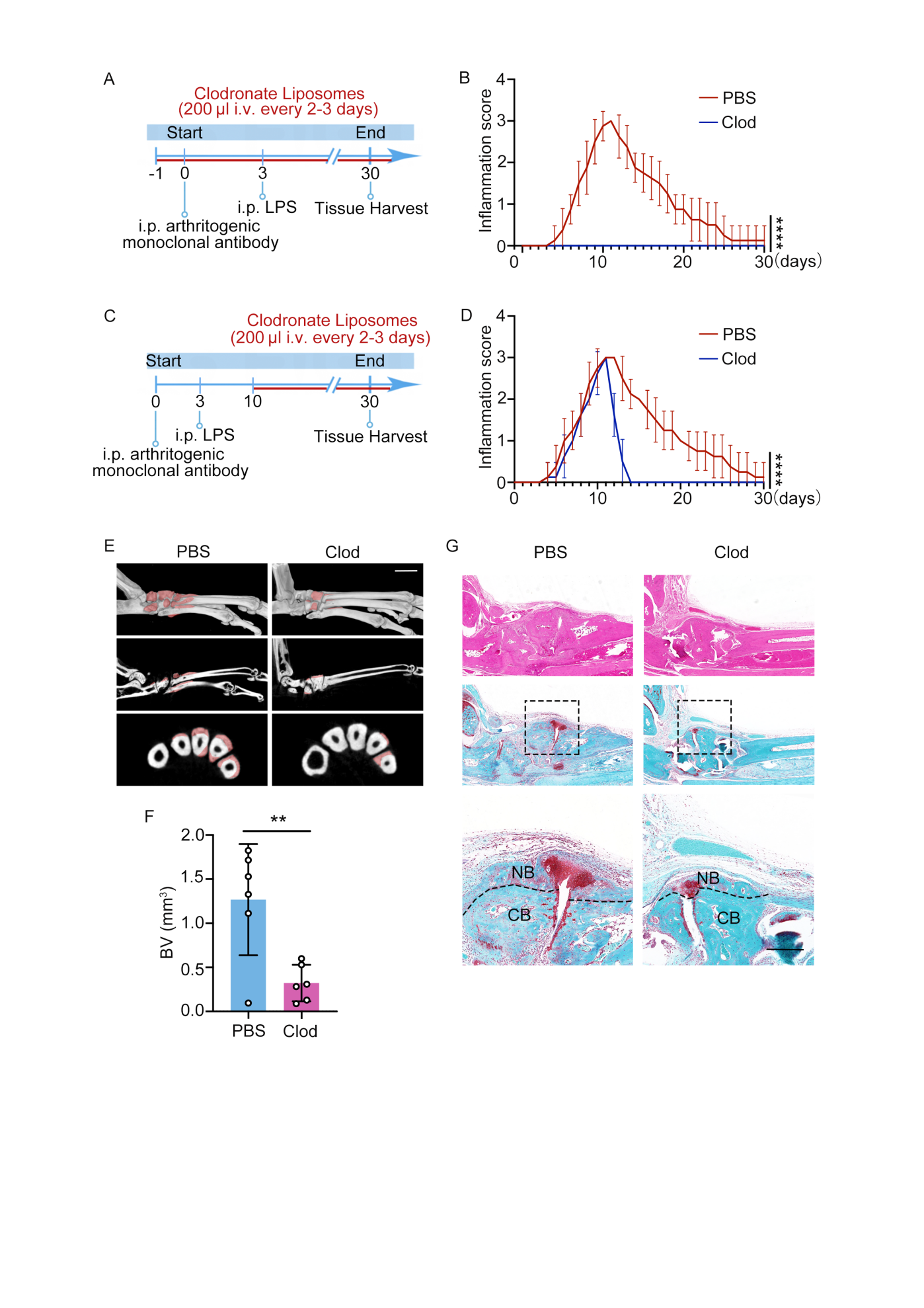


**Figure S2.** (**A**) An illustration of induction and intervention of CAIA model. (**B**) Inflammation score of CAIA model. n=6, repeated measurements with generalised estimating equations, data shown as mean±SD. (**C**) An illustration of induction and intervention of CAIA model. (**D**) Inflammation score of CAIA model. n=6, repeated measurements with generalised estimating equations, data shown as mean±SD. (**E** and **F**) μCT images and quantitative analysis of pathological new bone formation in PBS and clodronate-treated CAIA model. n = 6 per group. Scale bar: 1.5 mm. (**G**) H&E staining and SOFG staining in hind paws of PBS and clodronate-treated CAIA model. Scale bar: 200 µm. Data shown as mean±SD. **p<0.01; ****p<0.0001; determined by unpaired, two-tailed Student’s t-test. AS, ankylosing spondylitis; Ctrl, control; Cold, clodronate; BV, bone volume; CB, cortical bone; NB, new bone.

**
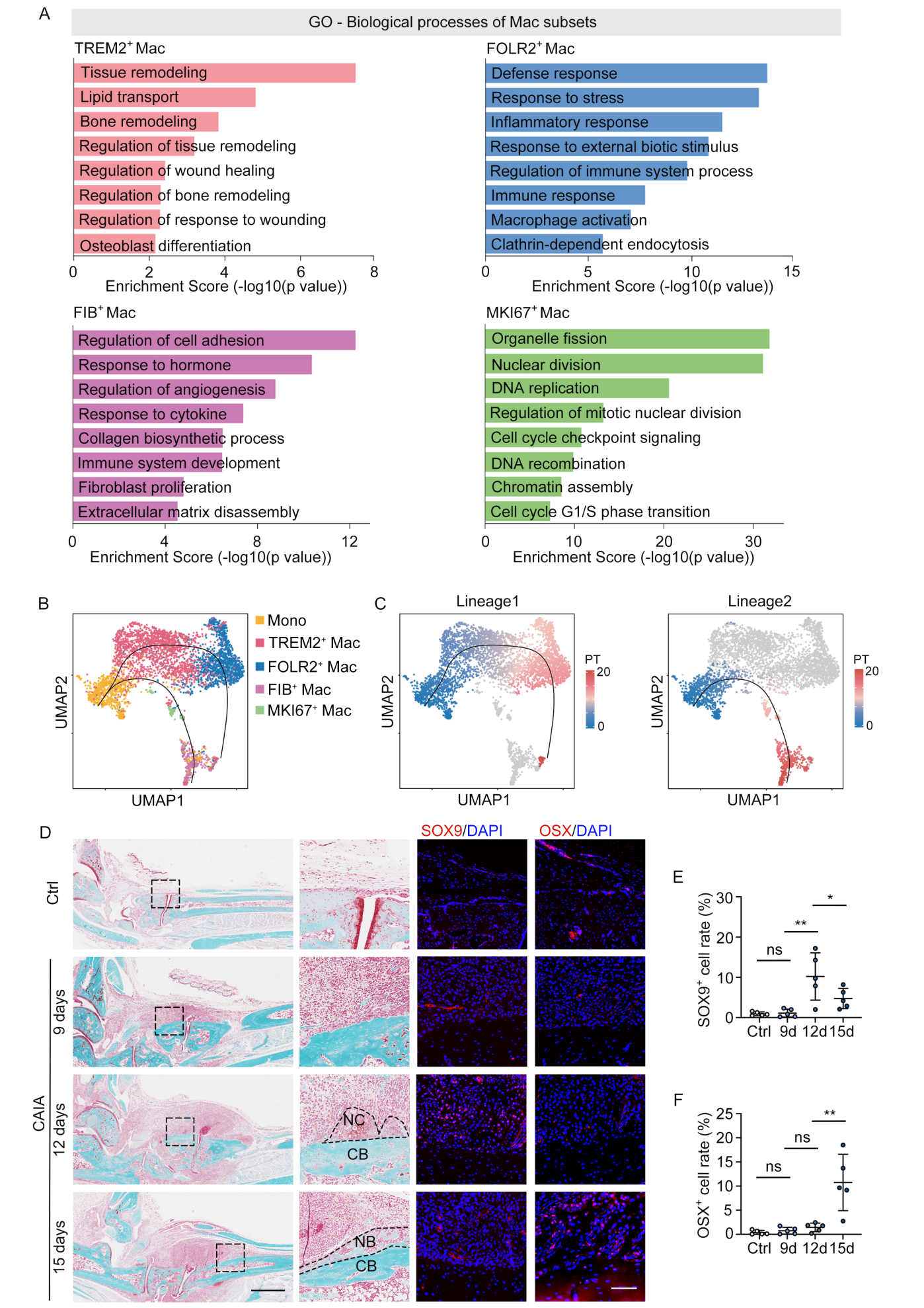
**

**Figure S3.** (**A**) GO pathway analysis of upregulated genes in each macrophages subsets of scRNA-seq. (**B** and **C**) UMAP plot of pseudotime analysis of subsets monocytes, Trem2+ macrophages,Folr2+ macrophages,Fib+ macrophages and Mki67+ macrophages. (**D**) SOFG staining and IF staining in hind paws of CAIA model, including staining for SOX9 and OSX. n = 5 per group. Scale bar: 200 µm. (**E** and **F**) Quantitative analysis of D. One-way ANOVA with Tukey’s post hoc test. Data shown as mean±SD. *p<0.05; **p<0.01; ns, not significant (P > 0.05). CAIA, collagen antibody-induced arthritis; Ctrl, control; NC, new cartilage; CB, cortical bone; NB, new bone.

**
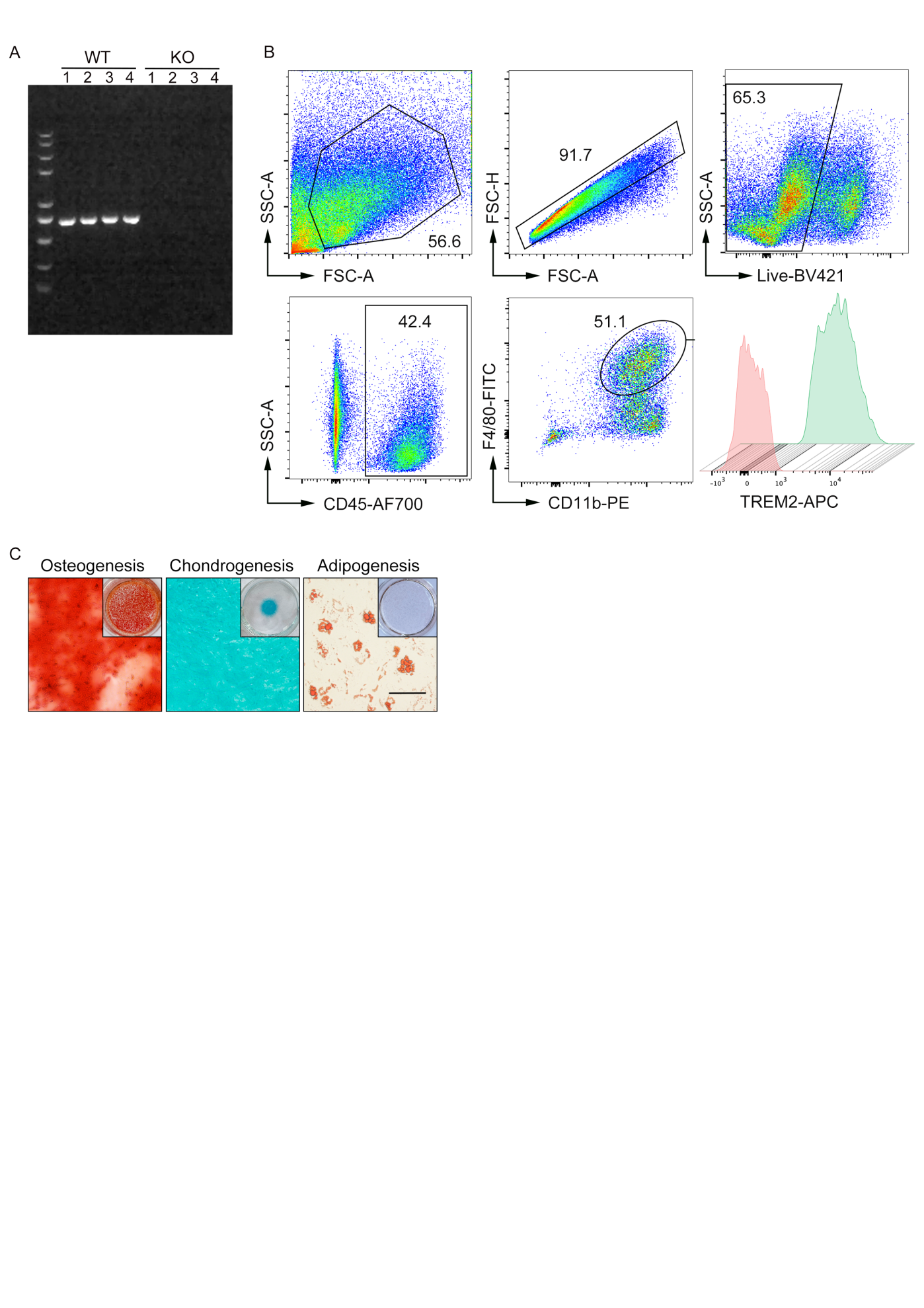
**

**Figure S4.** (**A**) Gel electrophoresis results of the PCR amplifications of RNA from WT mice and KO mice. (**B**) Gating strategy for flow cytometry analysis and flow sorting and collection of Trem2^hi^ macrophage. (**C**) Alizarin red, alcian blue and oil red O staining of ligament-derived progenitor cells under osteogenesis, chondrogenesis or adipogenesis. n=3. Scale bar: 20μm.

**
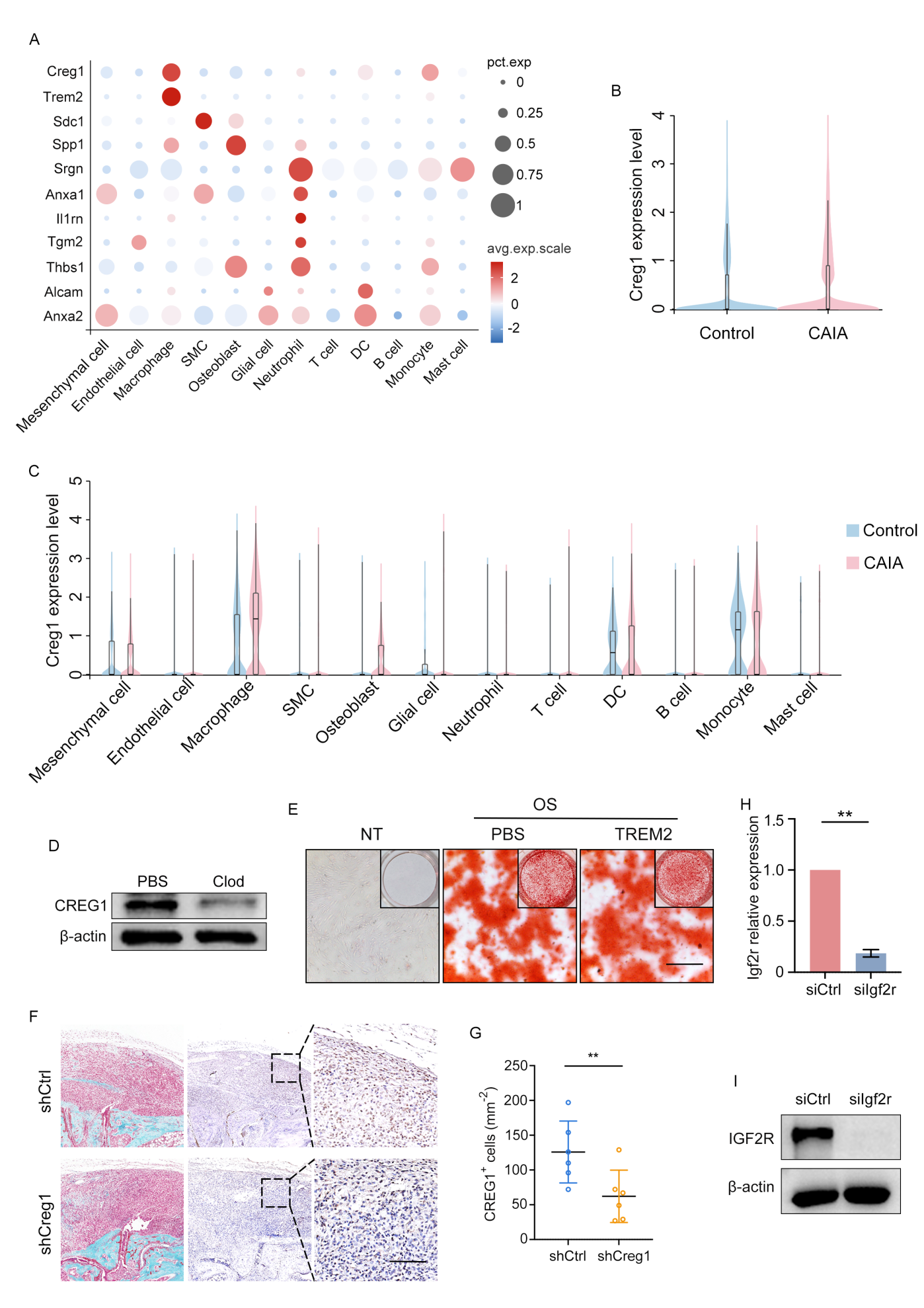
**

**Figure S5.** (**A**) Bubble diagram of candidate secretory protein genes in each cluster. (**B**) Violin plots of Creg1 expression in control and CAIA groups. (**C**) Violin plots of Creg1 expression across all cell types in both the control and CAIA datasets. (**D**) Immunoblot analysis of CREG1 levels in PBS and clodronate treated CAIA model. (**E**) Alizarin Red staining of LDPCs treatment with TREM2 for 14 days. n=3. Scale bar: 100 µm. (**F**) SOFG staining, immunohistochemical analysis of CREG1 in hind paws of CAIA model with administration of shCtrl or shCreg1 for 12 days. n = 6 per group. Scale bar: 100 µm. (G) Quantitative analysis of F. (**H** and **I**) RT-qPCR analysis and immunoblot analysis of the level of IGF2R in BMSCs with knockdown of IGF2r. Data shown as mean±SD. **p<0.01 determined by unpaired, two-tailed Student’s t-test. AS, ankylosing spondylitis.

**
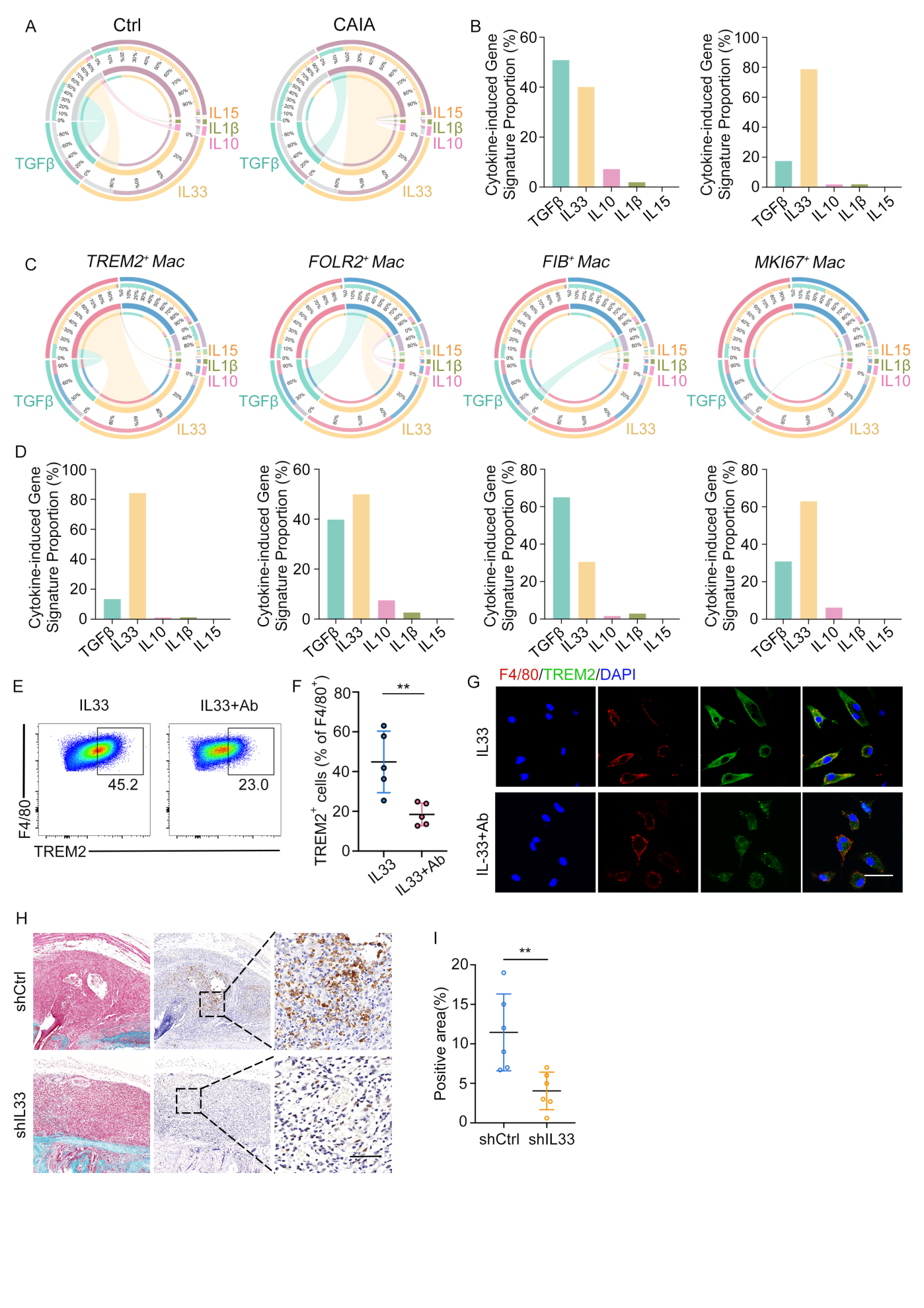
**

**Figure S6.** (**A**) Circos plots shows the enrichment score of cytokine-induced gene set in macrophages from both the control and the CAIA model. (**B**) Quantitative analysis of A. (**C**) Circos plots shows the enrichment score of cytokine-induced gene signature in TREM2^+^ macrophages, FOLR2^+^ macrophages, FIB^+^ macrophages and MKI67^+^ macrophage. (**D**) Quantitative analysis of C. (**E**) Flow cytometry analysis of Trem2 expression in IL-33–induced macrophages from BMDMs with or without ST2 neutralizing antibody. n = 5 per group. (**F**) Quantitative analysis of B. (**G**) Immunocytochemical staining of BMDMs by Trem2 and F4/80 after IL-33 treatment with or without ST2 neutralizing antibody for 3 days (shown is one representative result from n = 5 BRs). (H) SOFG staining, immunohistochemical analysis of IL-33 in hind paws of CAIA model with administration of shCtrl or shIL-33 for 12 days. n = 6 per group. Scale bar: 100 µm. (I) Quantitative analysis of H. Scale bar: 20 µm. Data shown as mean±SD. **p<0.01 determined by unpaired, two-tailed Student’s t-test. Ab, ST2 neutralizing antibody.

**
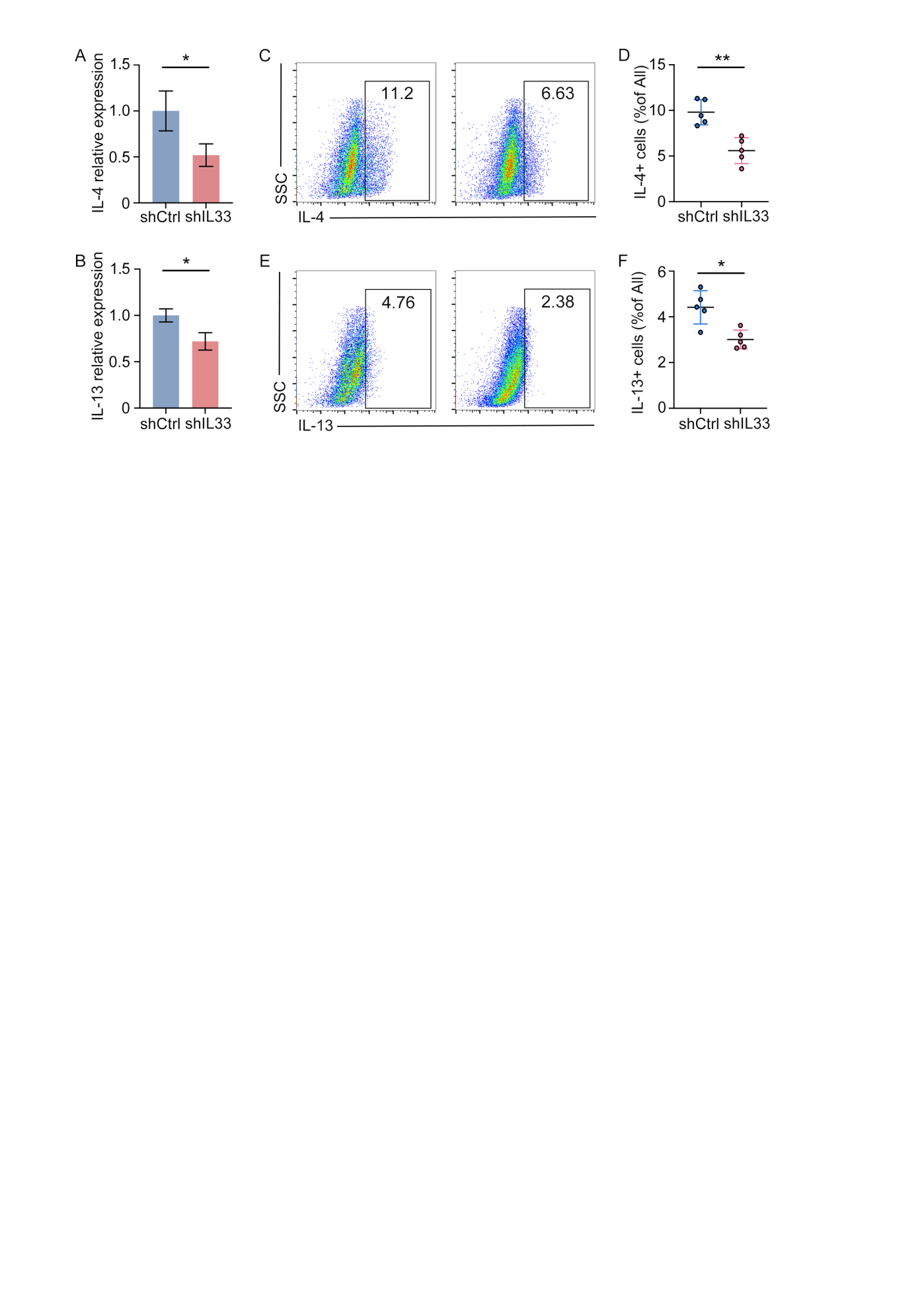
**

**Figure S7.** (**A**) RT-qPCR analysis of the level of IL-4 in CAIA model with administration of shCtrl or shIL-33 for 10 days. (**B**) RT-qPCR analysis of the level of IL-13 in CAIA model with administration of shCtrl or shIL-33 for 10 days. (**C**) Flow cytometry analysis of IL-4+ cells in CAIA model with administration of shCtrl or shIL-33 for 10d. (**D**) Quantitative analysis of IL4+ cells in (C). n = 5 per group. (**E**) Flow cytometry analysis of IL-13+ cells in CAIA model with administration of shCtrl or shIL-33 for 10d. (**F**) Quantitative analysis of IL13+ cells in (E). n = 5 per group. *p<0.05; **p<0.01 determined by unpaired, two-tailed Student’s t-test.

**Table1 S1.** Screening for Secreted Proteins

| **Secreted proteins in the AS group** | **Secreted proteins in TREM2^+^ macrophages** | **Intersection of secreted proteins in the AS group and those in TREM2^+^ macrophages** |
| --- | --- | --- |
| CFH | GRN | CREG1 |
| SERPINB1 | TREM2 | IL1RN |
| FAS | LGALS1 | ITM2B |
| TLL1 | SPP1 | NRG1 |
| VCAN | APOE | SELENOP |
| ADAM28 | GDF15 | SPP1 |
| CHRDL2 | LGALS3 | TGM2 |
| COL11A1 | LAMC1 | TREM2 |
| FGFR2 | ITM2B |  |
| FGFR3 | IL1RN |  |
| GAL | CREG1 |  |
| NUCB2 | ECM1 |  |
| EDN1 | NRG1 |  |
| PCDHA6 | CXCL16 |  |
| C1QTNF3 | CTSB |  |
| PIBF1 | LPL |  |
| TXNDC16 | FAM20C |  |
| NID2 | PDGFA |  |
| PTHLH | TGM2 |  |
| LYZ | TNFSF12 |  |
| CMA1 | SELENOP |  |
| TGFB2 |  |  |
| TREM2 |  |  |
| COCH |  |  |
| MXRA5 |  |  |
| CD40LG |  |  |
| TIMP1 |  |  |
| TNFSF13B |  |  |
| CRISPLD2 |  |  |
| CEMIP |  |  |
| SCG3 |  |  |
| CCN4 |  |  |
| IL4I1 |  |  |
| CLEC11A |  |  |
| NAMPT |  |  |
| PTN |  |  |
| WNT2 |  |  |
| NPTX2 |  |  |
| PTPRZ1 |  |  |
| OGN |  |  |
| ASPN |  |  |
| ECM2 |  |  |
| WNT3 |  |  |
| CCL2 |  |  |
| COL1A1 |  |  |
| CPZ |  |  |
| MDK |  |  |
| COL12A1 |  |  |
| LOX |  |  |
| GZMK |  |  |
| SPARC |  |  |
| EFEMP1 |  |  |
| IGFBP2 |  |  |
| IL1R1 |  |  |
| IL1RL1 |  |  |
| SDC1 |  |  |
| PAPPA2 |  |  |
| OLFML3 |  |  |
| BMP8B |  |  |
| CD48 |  |  |
| C1orf54 |  |  |
| CCN2 |  |  |
| SPP1 |  |  |
| TGFB3 |  |  |
| TEK |  |  |
| PLAU |  |  |
| SRGN |  |  |
| COL10A1 |  |  |
| NMI |  |  |
| TREM1 |  |  |
| COL21A1 |  |  |
| EREG |  |  |
| OMD |  |  |
| LIF |  |  |
| LRRC17 |  |  |
| TWSG1 |  |  |
| OLFM1 |  |  |
| COL5A1 |  |  |
| ULBP2 |  |  |
| MATN3 |  |  |
| CHI3L1 |  |  |
| POSTN |  |  |
| C1QTNF6 |  |  |
| NTS |  |  |
| IL6ST |  |  |
| FST |  |  |
| ANXA1 |  |  |
| SERPINE2 |  |  |
| ITM2B |  |  |
| IL6 |  |  |
| IL1RN |  |  |
| ENPP2 |  |  |
| CCN3 |  |  |
| IL33 |  |  |
| IGFBPL1 |  |  |
| KIAA0319 |  |  |
| PI15 |  |  |
| THBS1 |  |  |
| CGREF1 |  |  |
| MMRN1 |  |  |
| FGF7 |  |  |
| ADAMTS18 |  |  |
| CREG1 |  |  |
| HMCN1 |  |  |
| GDF7 |  |  |
| COL8A1 |  |  |
| PLA1A |  |  |
| UCN2 |  |  |
| MANF |  |  |
| SFRP2 |  |  |
| SCUBE3 |  |  |
| ADAM12 |  |  |
| IL18 |  |  |
| FAM177A1 |  |  |
| SPOCK1 |  |  |
| IGSF10 |  |  |
| LGI2 |  |  |
| PLA2R1 |  |  |
| LY96 |  |  |
| CXADR |  |  |
| ADAMTS1 |  |  |
| DKK2 |  |  |
| MMP16 |  |  |
| ADAMTS3 |  |  |
| NRG1 |  |  |
| ADAMTS4 |  |  |
| OLFML2B |  |  |
| IGFBP7 |  |  |
| SERPINI1 |  |  |
| ADAMTS9 |  |  |
| CXCL5 |  |  |
| CPA3 |  |  |
| AIMP1 |  |  |
| IL15 |  |  |
| EDIL3 |  |  |
| ARSK |  |  |
| ERAP2 |  |  |
| COL1A2 |  |  |
| FNDC1 |  |  |
| SBSPON |  |  |
| CTHRC1 |  |  |
| OTOGL |  |  |
| JAM3 |  |  |
| B2M |  |  |
| C18orf54 |  |  |
| SERPINF2 |  |  |
| COL3A1 |  |  |
| ADAM9 |  |  |
| NPNT |  |  |
| CXCL8 |  |  |
| ALCAM |  |  |
| MZB1 |  |  |
| PDGFD |  |  |
| SOSTDC1 |  |  |
| SCG2 |  |  |
| CLCF1 |  |  |
| ADAMTSL1 |  |  |
| ARSJ |  |  |
| TNFSF15 |  |  |
| C1S |  |  |
| IGIP |  |  |
| ANXA2 |  |  |
| CCBE1 |  |  |
| BMP8A |  |  |
| SLIT3 |  |  |
| MUC1 |  |  |
| NTF3 |  |  |
| NRG3 |  |  |
| THBS2 |  |  |
| COL14A1 |  |  |
| FAM180A |  |  |
| HMGB1 |  |  |
| IL1RAP |  |  |
| MMP1 |  |  |
| FAM3C |  |  |
| PDCD1LG2 |  |  |
| TGM2 |  |  |
| IGKV3-20 |  |  |
| IGKV3-11 |  |  |
| CFB |  |  |
| CFHR1 |  |  |
| IGKV3-15 |  |  |
| SELENOP |  |  |
| HP |  |  |
| SPESP1 |  |  |
| GDF10 |  |  |

**Table1 S2.** Cytokines-induced gene signatures.

| IL33_GSE43660_Eosinophils_1h | IL1_GSE8515_Monoctye_6h | IL6_GSE8515_Monoctye_6h | Il10_GSE59184_Monocyte_6h | Il15_GSE59184_Monocyte_6h | TGFb_GSE48579_Microglia |
| --- | --- | --- | --- | --- | --- |
| Nfkbiz | Abca1 | Acvr2b-As1 | Abhd17c | Csf3 | Sall1 |
| Med16 | Actl6b | Ankrd26 | Acap1 | Il6 | Bhlhe41 |
| Cdc42ep2 | Adra1a | Ankrd53 | Acpp | C1qtnf1 | Gtf2h2 |
| Cd69 | Aff3 | Apoa1 | Adcy1 | Flt1 | Bin1 |
| Cxcl2 | Agrp | Areg | Adgra2 | Ebi3 | Myo1b |
| Nfkbiz | Agtr1 | Armc9 | Agfg1 | Has1 | Bco2 |
| Tnf | Alox15 | Atp2b2 | Amigo2 | Ccl20 | Ltc4s |
| Cxcl10 | Ambp | Atp8a2 | Angptl4 | Gjb2 | Spsb1 |
| Tnfaip3 | Angptl3 | C10orf10 | Anxa3 | Tfpi2 | Hps4 |
| Il6 | Ankrd26 | C14orf1 | Apcdd1 | Il12b | Golm1 |
| Zc3h12a | Apba3 | C5orf45 | Aqp3 | Tnip3 | St3gal6 |
| Adora2b | Apoa1 | C9 | Arhgap12 | Ndp | X99384 |
| Irg1 | Apol5 | Cabp5 | Arnt2 | F3 | B4galt4 |
| Dusp8 | Appbp2 | Cc2d1a | Asph | Itgb8 | Ak1 |
| Smpdl3b | Arl17b | Ccdc181 | Atp2b4 | Il36g | Arhgap5 |
| Nfkbia | Asap2 | Cd14 | Avpr1a | Mir3945hg | Tmem195 |
| Nr4a3 | Asb6 | Cd22 | B3gnt7 | Inhba | Ctsf |
| Tnfaip3 | Atp8a2 | Cdkn2a-As1 | Bank1 | Il2ra | Rilpl1 |
| Psd | Bid | Ces1p1 | Batf | Klhl14 | Hexb |
| Icam1 | Btd | Col14a1 | Batf3 | Acod1 | Cryl1 |
| Ptgs2 | Btg3 | Col5a2 | Bcl6 | Sgpp2 | Usp2 |
| Gem | C14orf1 | Col6a1 | C10orf10 | Il23a | Mlph |
| Serpine1 | C3orf52 | Coq8b | C1orf21 | Hspb7 | Fscn1 |
| Ccrl2 | Cabp5 | Cps1-It1 | C1qc | Slco4a1 | Fgd2 |
| Pde4b | Cand1.11 | Crct1 | C1rl | Dll1 | Sparc |
| Cish | Caskin2 | Csdc2 | C3orf70 | Wfdc21p | Olfml3 |
| Traf1 | Cass4 | Cyp3a43 | C6orf223 | Pld1 | Adamts16 |
| Pde4b | Cc2d1a | Dbh | Carmil1 | Mapkapk5-As1 | Pdgfa |
| Nfkbie | Ccdc28b | Dlg4 | Ccdc190 | Actrt2 | Plxdc2 |
| Ralgds | Ccl20 | Dzip1 | Ccl18 | Ebf1 | Cst3 |
| Niacr1 | Ccl3l3 | Dzip3 | Ccl8 | Loc100507855 | Serpine2 |
| Clec4e | Ccl4 | Egfr | Ccm2l | Tnfsf15 | Pdgfb |
| Il13 | Ccr7 | Elovl4 | Cd163 | Rasgrp1 | P2ry12 |
| Bmp2 | Cd44 | Epha4 | Cd274 | Ankef1 | P2ry13 |
| Nfkbid | Cd6 | Fam174b | Cd300a | Ccl23 | Gpr34 |
| Rrad | Cd79b | Fam66d | Cd47 | Tnc | Csf3r |
| Il1rn | Cd84 | Fezf2 | Cdc14a | Cdk18 | Tlr3 |
| Slc2a6 | Celf1 | Foxn1 | Cdc42ep5 | Mir155 | Eng |
| Bcl3 | Celsr3-As1 | Gabbr2 | Cdk2 | Msantd3-Tmeff1 | Slc2a5 |
| Gadd45b | Cep97 | Galk1 | Cdkn2d | Ccl15-Ccl14 | Slco2b1 |
| Zfp295 | Cflar | Gh1 | Cemip | G0s2 | Slc24a3 |
| Nfkb2 | Chrne | Gpr1 | Cfap46 | Mcoln2 | Atp8a2 |
| Chuk | Chst2 | Gucy1b2 | Chchd7 | Plac8 | Kcnd1 |
| Il1b | Cnot4 | Gypb | Chi3l2 | Scn1b | Tgfbr1 |
| Vcam1 | Col3a1 | H2afb1 | Clec4gp1 | Stat4 | Tgfbr2 |
| Cxcl3 | Crct1 | H2afx | Cngb1 | Il1a | Siglech |
| Gtf2ird2 | Cxcl1 | Hnf4a | Cpm | C1orf61 | Tmem119 |
| Rab20 | Cxcl2 | Hnf4g | Cr1 | Elovl7 | Cd34 |
| Dusp2 | Cxcl3 | Hp1bp3 | Cr1l | Cd38 | F11r |
| Relb | Cxcl5 | Htr2c | Crem | Loc100506098 | Fcrls |
| Prkab2 | Cxcl6 | Ifna13 | Cryaa | Msantd3 | Lrrc3 |
| Il1a | Cxcl8 | Ifna21 | Csrp2 | Abca8 | Ppp1r9a |
| Pilrb2 | Ddit4 | Igf2-As | Ctslp8 | Baiap2l1 | Rtn4rl1 |
| Sec24a | Dlg4 | Ighm | Ctsv | Dnaaf1 | Jam2 |
| Rab11fip1 | Dnajb5 | Igkc | Cx3cr1 | C11orf96 | Cxxc5 |
| Il10ra | Dux1 | Il12rb1 | Cxcl13 | Palm2-Akap2 | Tspan7 |
| Sod2 | Dzip1 | Il6r | Cyth4 | Ccl1 | Pmepa1 |
| Bcl2a1a | Dzip3 | Itpka | Ddx28 | Kiaa1211 | Itgb5 |
| Pim3 | Efna3 | Itsn1 | Dennd2d | Scg5 | Tmem100 |
| Zc3h12c | Egfr | Kcna4 | Dll1 | Mmp14 |  |
| Nod2 | Ehd1 | Kcng1 | Dsc2 | Ptgs2 |  |
| Bcl2a1d | Eif5a2 | Kdm8 | Eda | Cdc42ep5 |  |
| Nfkb2 | Eif5b | Klf8 | Ednrb | Loc440934 |  |
| Dusp16 | Elovl2 | Klk12 | Ehd1 | Stk26 |  |
| Chka | Emx1 | Krt16 | Enpp2 | Serpinb9 |  |
| Jun | Fabp6 | Loc100996693 | Epha2 | Mamld1 |  |
| Zfp568 | Fam174b | Loc101926997 | Etv5 | Hey1 |  |
| Zfp599 | Fer1l4 | Loc101929540 | F13a1 | Pkig |  |
| Bcl2l11 | Fgf5 | Mal | F5 | Il15ra |  |
| Itpkc | Foxn1 | Maob | Fam20a | Upb1 |  |
| Cdk19 | Fut4 | Map3k19 | Fam72a | Enpp2 |  |
| Nr4a1 | G0s2 | Mapk11 | Fcgr1a | Ctag1a |  |
| Pim2 | Gabbr2 | Mast4 | Fcgr1b | Lamp3 |  |
| Ier3 | Gadd45b | Med16 | Fcgr1cp | C1orf21 |  |
| Fosl1 | Gap43 | Med27 | Fcrlb | Cxcl5 |  |
| Dom3z | Gli1 | Meg3 | Ffar2 | Serpinb9p1 |  |
| Ect2 | Gnal | Mettl10 | Fga | Parp15 |  |
| Gpr84 | Gpr132 | Mlip | Fhl1 | Mfsd2a |  |
| Trpc4ap | Gpr18 | Muc5ac | Fjx1 | Cemip |  |
| Slc29a2 | Grik2 | Mycnos | Flot2 | Socs1 |  |
| Rel | Grk2 | Myh4 | Flt1 | Chi3l1 |  |
| Maff | Hcar3 | Myod1 | Fnip2 | Pdgfrl |  |
| Bcl2a1b | Hck | Nav3 | Fpr1 | Gng2 |  |
| Pim1 | Hnf4a | Nefl | Fpr2 | Tnfrsf4 |  |
| Cyld | Hook1 | Nek1 | Fzd2 | Rhobtb3 |  |
| Il4 | Hoxb7 | Nfib | Fzd5 | Kcne5 |  |
| Map3k8 | Huwe1 | Nlgn4y | Gadd45b | Tcea3 |  |
| Tnfaip2 | Icam1 | Nphs1 | Gas6 | Pde4b |  |
| Tcfe3 | Ier3 | Nrxn3 | Ggn | Gzmb |  |
| Tgif2 | Ighv3-23 | Nxf2b | Ghrhr | Edrf1 |  |
| F10 | Iglv1-44 | Or2j3 | Gng2 | Amigo2 |  |
| Rab38 | Il6 | Pard3 | Gpr85 | Hdac9 |  |
| Ccl4 | Il7r | Pax2 | Gzmb | Loc100129518 |  |
| Spata13 | Inpp5j | Phox2a | Hamp | Ccdc178 |  |
| Xkr8 | Jag1 | Pidd1 | Has1 | Gbp1p1 |  |
| Gpr132 | Kcnn3 | Ppial4c | Heg1 | Batf |  |
| Mapk6 | Kcnv1 | Ppp4r4 | Hes1 | Il7r |  |
| Dyrk3 | Krt84 | Prim2b | Hfe | Ido1 |  |
| Adora2a | Krtap2-3 | Prmt2 | Hivep2 | Rhbdl2 |  |
| Slc25a33 | Lad1 | Ptger3 | Hmgn5 | Gadd45b |  |
| Ets2 | Lif | Ptpn1 | Hoxb8 | Ddr1-As1 |  |
| Grinl1a | Lifr | Pyy | Hpgd | Arnt2 |  |
| Tlr2 | Limk2 | Rbpms | Htra4 | Nrp2 |  |
| Peg10 | Lman1l | Rergl | Ibsp | Cfap46 |  |
| Ifrd1 | Loc100129518 | Rit2 | Ifi16 | Il32 |  |
| Suz12 | Loc100996693 | Sbspon | Ifitm2 | Loc399716 |  |
| Igsf6 | Loc100996792 | Scgb2a2 | Ifitm3 | Cd274 |  |
| Tbc1d24 | Loc101928457 | Sec24d | Ikzf2 | Met |  |
| Dem1 | Loc101930405 | Sept5-Gp1bb | Il10 | Grin1 |  |
| Rnd1 | Lrit1 | Sfrp1 | Il1rn | Il7 |  |
| Phc3 | Lrrc32 | Sftpb | Il21r | Ffar2 |  |
| Junb | Lsm4 | Slc17a6 | Il7 | Kcnj2-As1 |  |
| Pilrb1 | Magea1 | Slc22a6 | Il7r | Lsm11 |  |
| Bcl2l11 | Magea9b | Slc2a3 | Inhbb | Wnt5a |  |
| Nlrp3 | Map3k8 | Slc6a2 | Jak3 | Slamf7 |  |
| Phlda1 | Mboat7 | Snora70 | Jrk | Ptn |  |
| Pdlim7 | Mir6732 | Socs3 | Kiaa0226l | Hs3st3b1 |  |
| Ralgds | Mir6758 | Spaca1 | Kiaa1211l | Zmiz1-As1 |  |
| Cxcl1 | Mir8085 | Spag11a | Kremen1 | Yme1l1 |  |
| Birc3 | Mmp15 | Spdef | Lilra1 | Mt2a |  |
| Asb2 | Mog | Ssh3 | Lilra3 | Fpr2 |  |
| Pde2a | Msc | Ssx2ip | Lilra5 | Loc101927811 |  |
| Egr3 | Mtf1 | St6gal1 | Lilrp2 | Pdgfa |  |
| Fnbp1 | Myh14 | Susd5 | Limk2 | Mt1g |  |
| Nfkbib | Nck2 | Synj2 | Linc00189 | Fez1 |  |
| Kcnj2 | Nebl | Tbc1d29 | Linc01093 | Pim1 |  |
| Ell2 | Nek1 | Tgm4 | Linc01128 | Vnn3 |  |
| Ppp1r15a | Nell2 | Tmem100 | Litaf | Hspb3 |  |
| Plk2 | Nf2 | Tmem2 | Lmnb1 | Actl7a |  |
| Marcksl1 | Nfkb1 | Trim29 | Loc100130468 | Tcl6 |  |
| Ltb | Nfkb2 | Tspy1 | Loc101926921 | Map3k4 |  |
| Ptgir | Nfkbia | Tsr3 | Loc101926963 | Etv5 |  |
| Afg3l1 | Nfkbib | Ttc38 | Loc101927503 | Mt1x |  |
| Gm614 | Nfkbie | Ucp3 | Loc101927507 | Mreg |  |
| Il1rn | Nid1 | Uts2 | Loc101928144 | Plau |  |
| Metrnl | Nr1d1 | Vsx1 | Loc101928635 | Gucy1a2 |  |
| Atg16l2 | Nr5a2 | Znf287 | Loc101929459 | Lrig1 |  |
| Socs3 | Osgin2 | Znf446 | Loc105372547 | Cd80 |  |
| Camta2 | Ovol2 | Znf460 | Loc106146153 | Ctslp8 |  |
| Dom3z | Pak6 | Znf771 | Loc154761 | Unc13d |  |
| Gaa | Pax2 | Zswim8 | Loc285957 | Rin2 |  |
| Raly | Pdcd2 |  | Loc399716 | Cyp3a5 |  |
| Gcnt1 | Plk3 |  | Loc441081 | Rhof |  |
| Stx11 | Pml |  | Lrg1 | Map3k5 |  |
| Ccl3 | Pnliprp1 |  | Lrp5 | Ccl8 |  |
| Arl5b | Pnrc1 |  | Map3k6 | Kcnk1 |  |
| Olr1 | Ppif |  | Mapk11 | Tnfrsf18 |  |
| Socs2 | Prkaca |  | Marco | Specc1l-Adora2a |  |
| Ubr5 | Ptger4 |  | Mctp2 | Loc101929500 |  |
| Klf10 | Ptx3 |  | Megf6 | Thnsl1 |  |
| Six5 | Pvr |  | Mgat4a | Abhd17c |  |
| Gm12505 | Pycrl |  | Mir1182 | Cyb5r2 |  |
| P2ry13 | Rab27b |  | Mir1247 | Ece1 |  |
| Gabpb2 | Ranbp1 |  | Mir3945hg | Pnpla1 |  |
| Col1a1 | Rbpms |  | Mmp12 | Exosc10 |  |
| N4bp1 | Rel |  | Mob3b | Mt1f |  |
| Rbm12 | Relb |  | Mov10l1 | Socs3 |  |
| Rfx5 | Rere |  | Moxd1 | Rgl4 |  |
| Ddx6 | Rgs5 |  | Ms4a4a | Trpm1 |  |
| Zfp869 | Sbspon |  | Ms4a6a | Shc3 |  |
| Casp4 | Sdc4 |  | Mucl1 | Slamf1 |  |
| Clec4n | Sftpb |  | Mxd1 | Loc101930416 |  |
| Prr7 | Sik3 |  | Myo7a | Lrrc17 |  |
| Homez | Slc11a2 |  | Myog | Gys2 |  |
| Ier5 | Slc16a4 |  | Myt1l | Ror1 |  |
| Rab11fip1 | Slc1a2 |  | Nfe2 | Miip |  |
| Bcl2a1c | Slc26a3 |  | Nheg1 | Fgf2 |  |
| Prr3 | Slc2a6 |  | Nkg7 | Rnd1 |  |
| Sin3a | Slc37a1 |  | Olfml2b | Rnf144b |  |
| Fam110a | Slc39a8 |  | Otud1 | Ehd1 |  |
| Extl2 | Slc4a4 |  | P2rx2 | Dlgap1-As2 |  |
| Chka | Slc6a9 |  | P2ry2 | Linc01093 |  |
| Mecp2 | Smox |  | Pcolce2 | Linc01215 |  |
| Gbp2 | Snn |  | Pdcd1lg2 | Fam65b |  |
| Ggta1 | Spaca1 |  | Pdgfa | Nbpf20 |  |
| Egr2 | Specc1l-Adora2a |  | Pdpn | Mt1hl1 |  |
| Col6a2 | Sspn |  | Pim1 | Ccl2 |  |
| Mtm1 | Stat4 |  | Plekhg2 | Espn |  |
| Arhgap4 | Stx11 |  | Plgrkt | C22orf42 |  |
| Gbp1 | Suv39h2 |  | Plscr4 | Shox |  |
| Kat5 | Tbx5 |  | Ppp1r3b | Mgst3 |  |
| Pls3 | Tcl6 |  | Prkch | Arl5b |  |
| 2310014H01Rik | Tfr2 |  | Prok2 | Snx9 |  |
| Sec22c | Tmem100 |  | Pros1 | Slc39a8 |  |
| Tgm2 | Tmsb4y |  | Prune2 | Znf782 |  |
| Tnip3 | Tnf |  | Ptpn2 | Loc105370943 |  |
| Gadd45a | Tnfaip3 |  | Ptx3 | Gramd1a |  |
| Filip1l | Tnfaip6 |  | Rab42 | Ppp4r4 |  |
| Srrt | Tnfrsf9 |  | Ralgps2 | Loc84214 |  |
| Tmem39a | Tnip1 |  | Rassf4 | Serpinb7 |  |
| Rnf19b | Tnp2 |  | Ren | Pou5f1b |  |
| Cdk6 | Tpsb2 |  | Rhbg | Sh3bgr |  |
| Cant1 | Traf1 |  | Rhobtb3 | Mt1e |  |
| Tgif1 | Trip10 |  | Rnf157 | Mt1h |  |
| Col3a1 | Trpm6 |  | S1pr1 | Il1rn |  |
| Fosb | Whrn |  | Sbno2 | Abcb5 |  |
| Tgif1 | Wnt5a |  | Sh2d3a | Bcl2a1 |  |
| Mllt6 | Wsb1 |  | Sh3pxd2b | Kcnj2 |  |
| Zfp429 | Xcl1 |  | Shb | Thsd7a |  |
| Mfsd6l | Yrdc |  | Shisa3 | Ccl7 |  |
| Asprv1 | Zbtb7b |  | Slamf1 | Loc100288911 |  |
| Myst2 | Znf480 |  | Slc11a1 | Bsnd |  |
| Mapk6 | Znf532 |  | Slc16a10 | Ccl5 |  |
| 5430427O19Rik | Znf771 |  | Slc16a7 | Tbc1d30 |  |
| Skil | Zscan12 |  | Slc1a2 | Loc101929459 |  |
| Zfp36l1 |  |  | Slc1a3 | Avpr1a |  |
| Tlr9 |  |  | Slc25a37 | C8orf74 |  |
| Jmjd4 |  |  | Slc2a14 | Ankrd1 |  |
| Egr1 |  |  | Slc2a3 | Xcl2 |  |
| Adam17 |  |  | Slco4a1 | Gzma |  |
| Jup |  |  | Smagp | Uxs1 |  |
| Txnl4a |  |  | Socs1 | Smad2 |  |
| Mfsd7a |  |  | Socs3 | Cyb5d1 |  |
| Cyth1 |  |  | Spanxa2-Ot1 | Adtrp |  |
| Mreg |  |  | Spp1 | Dusp16 |  |
| Skil |  |  | Steap3 | Trip10 |  |
| Mocs1 |  |  | Stmn3 | Pde4dip |  |
| Morf4l2 |  |  | Strip2 | Cog1 |  |
| Acin1 |  |  | Sucnr1 | Hs3st3a1 |  |
| Col6a1 |  |  | Tgfa | Slc30a4 |  |
| Hrh1 |  |  | Tgm2 | Klhl23 |  |
| Clk1 |  |  | Tifa | 3-Jan |  |
| Gabpb1 |  |  | Timp4 | L2hgdh |  |
| Tifa |  |  | Tlr7 | Bdnf |  |
| 9130008F23Rik |  |  | Tlr8 | Zpbp2 |  |
| Cldn1 |  |  | Tm4sf20 | Polr3e |  |
| Col1a2 |  |  | Tmem108 | Pnpla7 |  |
| Zc3h18 |  |  | Tmem173 | Loc340090 |  |
| Arid5a |  |  | Tmtc1 | Dll3 |  |
| Cd80 |  |  | Tnfrsf10c | Insm2 |  |
| Hmga1 |  |  | Tnfrsf8 | Limk2 |  |
| Mapkapk2 |  |  | Tnfsf18 | Phactr1 |  |
| St6galnac6 |  |  | Tnip3 | Rab20 |  |
| Opa3 |  |  | Tpt1-As1 | Klk13 |  |
| Lpp |  |  | Trim71 | Catsperg |  |
| Arf4 |  |  | Trip10 | Kank1 |  |
| Dnajb4 |  |  | Tspy1 | C1orf204 |  |
| Arhgef3 |  |  | Ugcg | Myo1b |  |
| Plxnb2 |  |  | Vmp1 | Mgc20647 |  |
| Myo1c |  |  | Vps9d1 | Trib3 |  |
| Fbxo34 |  |  | Zadh2 | Ctsv |  |
| Atp8a1 |  |  |  | Znf781 |  |
| Senp2 |  |  |  | Chac1 |  |
| Tank |  |  |  | Ifng |  |
| Uhrf1 |  |  |  | Tmem206 |  |
| Pfkm |  |  |  | Nheg1 |  |
| Etv3 |  |  |  | Gldn |  |
| Slc2a6 |  |  |  | Rgs5 |  |
| Ddit4 |  |  |  | Cxcl2 |  |
| Nbn |  |  |  | Tpd52 |  |
| Nxnl2 |  |  |  | Hes1 |  |
| Fam53b |  |  |  | Plpp3 |  |
| Mars |  |  |  | Slc35f4 |  |
| B3gnt2 |  |  |  | Ido2 |  |
| Nupr1 |  |  |  | Loc541472 |  |
| Ehd1 |  |  |  | Ptprj |  |
| Zfp275 |  |  |  | Gadd45a |  |
| Itih1 |  |  |  | Loc101928443 |  |
| Naip2 |  |  |  | Lhfpl1 |  |
| Rbms1 |  |  |  | Aspg |  |
| Rai12 |  |  |  | Tsc22d1 |  |
| Bgn |  |  |  | Wnk2 |  |
| Thbs2 |  |  |  | Loc105373418 |  |
| Trex1 |  |  |  | Tmem44 |  |
| Zfp62 |  |  |  | Golga2p3y |  |
| Opa1 |  |  |  | Tacr1 |  |
| Gabpb1 |  |  |  | Rhbg |  |
| Serf1 |  |  |  | Flj35934 |  |
| Dusp16 |  |  |  | Gng11 |  |
| Gch1 |  |  |  | Odf3 |  |
| Phtf1 |  |  |  | Pim2 |  |
| Tyms |  |  |  | Wtap |  |
| St8sia4 |  |  |  | Ccl18 |  |
| Rassf4 |  |  |  | Tnfsf14 |  |
| Sgms2 |  |  |  | Celf4 |  |
| Arg2 |  |  |  | Sstr3 |  |
| Zfp275 |  |  |  | Stum |  |
| Dgcr2 |  |  |  | Cdh6 |  |
| Tle3 |  |  |  | Lta |  |
| Gbp3 |  |  |  | Enam |  |
| Itch |  |  |  | Cxcl6 |  |
| Icosl |  |  |  | Linc00705 |  |
| Zfp667 |  |  |  | Rab40c |  |
| Il16 |  |  |  | Loc644090 |  |
| Slc7a1 |  |  |  | Lilra5 |  |
| Tjap1 |  |  |  | Faf1 |  |
| Prkch |  |  |  | Gucy1a3 |  |
| Cd274 |  |  |  | Drc3 |  |
| Layn |  |  |  | Jrk |  |
| Arhgef10l |  |  |  | Lss |  |
| Ikbkg |  |  |  | Tnfaip6 |  |
| Cnot6l |  |  |  | Klrb1 |  |
| Olfr1033 |  |  |  | Ugt2b4 |  |
| Slc20a1 |  |  |  | Mybl2 |  |
| Cbfa2t2 |  |  |  | Mmp24 |  |
| Nab2 |  |  |  | Lcp2 |  |
| Psap |  |  |  | Fjx1 |  |
| Hmgcr |  |  |  | Ube2o |  |
| Plk4 |  |  |  | Tbc1d9 |  |
| Dtx4 |  |  |  | Rai2 |  |
| Ehmt1 |  |  |  | Asns |  |
| Zswim4 |  |  |  | Cfb |  |
| Dlg4 |  |  |  | Slc2a3 |  |
| Rap1gds1 |  |  |  | Clgn |  |
| Usp16 |  |  |  | Chdh |  |
| Casc4 |  |  |  | Tusc3 |  |
| Alas1 |  |  |  | Lrp6 |  |
| Cass4 |  |  |  | Ccl25 |  |
| Pag1 |  |  |  | Linc00323 |  |
| Eif4a2 |  |  |  | F7 |  |
| Golga2 |  |  |  | Cd44 |  |
| 2310016C08Rik |  |  |  | Cd22 |  |
| Csrnp1 |  |  |  | Prr16 |  |
| Bcl2l11 |  |  |  | Sorbs2 |  |
| Lancl1 |  |  |  | Plgrkt |  |
| Nudcd1 |  |  |  | Traf1 |  |
| Fip1l1 |  |  |  | Lamb3 |  |
| Mon2 |  |  |  | Asph |  |
| Fam49a |  |  |  | Cdk1 |  |
| Zfp672 |  |  |  | Hhip |  |
| Dyrk2 |  |  |  | Col27a1 |  |
| Slc25a25 |  |  |  | Il19 |  |
| Rbm7 |  |  |  | Hells |  |
| Ifnar1 |  |  |  | Iglc1 |  |
| Slc39a1 |  |  |  | Bbs1 |  |
| Nif3l1 |  |  |  | Loc374443 |  |
| Mink1 |  |  |  | Prss23 |  |
| Nfatc2 |  |  |  | Hrh1 |  |
| Klf2 |  |  |  | Foxp2 |  |
| Ccnl1 |  |  |  | Ptprs |  |
| Tmem87a |  |  |  | Batf3 |  |
| C5ar1 |  |  |  | Reck |  |
| Irf5 |  |  |  | Rras2 |  |
| Hnrnpf |  |  |  | Cd40 |  |
| Cdc14a |  |  |  | Retnlb |  |
| Insig1 |  |  |  | Ttll5 |  |
| Nfix |  |  |  | Rac2 |  |
| Atp7a |  |  |  | Magi2-As3 |  |
| Mapk8ip3 |  |  |  | Cdkl1 |  |
| Mid1 |  |  |  | Garnl3 |  |
| Ccl7 |  |  |  | Loc101927841 |  |
| Zfp62 |  |  |  | Grm1 |  |
| Ms4a7 |  |  |  | Grid2 |  |
| G0s2 |  |  |  | Dlgap1-As1 |  |
| Fam46a |  |  |  | Mgll |  |
| Lrrc49 |  |  |  | Gbp1 |  |
| Fam46c |  |  |  | Dlx2 |  |
| Zfp574 |  |  |  | Macc1 |  |
| Phf17 |  |  |  | Tysnd1 |  |
| Stk38l |  |  |  | Lgals4 |  |
| 1110028C15Rik |  |  |  | Smpd1 |  |
| Stau1 |  |  |  | C3orf18 |  |
| Ticam2 |  |  |  | Cenpu |  |
| Ankrd17 |  |  |  | Tifa |  |
| Tpbg |  |  |  | Syne4 |  |
| Nampt |  |  |  | Naaladl2-As3 |  |
| Ptafr |  |  |  | Loc100506895 |  |
| Msh5 |  |  |  | Cckbr |  |
| Ablim1 |  |  |  | Plat |  |
| Zfx |  |  |  | Wnt4 |  |
| Cxcl5 |  |  |  | Pvrl3-As1 |  |
| Slc24a1 |  |  |  | Hhla2 |  |
| Ctnnd1 |  |  |  | Fam24a |  |
| Plec |  |  |  | Smc5 |  |
| 5930434B04Rik |  |  |  | Clic4 |  |
| Ptp4a1 |  |  |  | Pik3ap1 |  |
| Pilra |  |  |  | St20 |  |
| Smox |  |  |  | Psat1 |  |
| Pde4b |  |  |  | Clec2d |  |
| Serpinb6a |  |  |  | Sspn |  |
| Mapk8ip3 |  |  |  | Mycn |  |
| Gm10584 |  |  |  | Hrh3 |  |
| Snx10 |  |  |  | Col4a3 |  |
| Zmiz2 |  |  |  | Edn1 |  |
| Rabgef1 |  |  |  | Mtss1 |  |
| Usp53 |  |  |  | C17orf96 |  |
| Cdkn1a |  |  |  | Loc101929255 |  |
| Grb10 |  |  |  | Leprot |  |
| Fbxl5 |  |  |  | Marcks |  |
| Txnrd1 |  |  |  | Sucnr1 |  |
| Hpn |  |  |  | Daz4 |  |
| Tnip1 |  |  |  | Nbn |  |
| Rdbp |  |  |  | Loc101926963 |  |
| Abcc4 |  |  |  | Tslp |  |
| Abl2 |  |  |  | Golga2p10 |  |
| Lpin1 |  |  |  | Fam129a |  |
| Vasn |  |  |  | Zc3h12c |  |
| Slc30a6 |  |  |  | Pou3f3 |  |
| Whsc1 |  |  |  | Tnik |  |
| Icmt |  |  |  | Slfn5 |  |
| Pou2f1 |  |  |  | Pmaip1 |  |
| Dclre1c |  |  |  | Icam1 |  |
| Cdk13 |  |  |  | Crim1 |  |
| Sfmbt1 |  |  |  | Mthfd2l |  |
| Aen |  |  |  | Itpr1 |  |
| Cd14 |  |  |  | Angptl4 |  |
| Mxd1 |  |  |  | Map4k4 |  |
| Leng9 |  |  |  | Mctp2 |  |
| Stx11 |  |  |  | Osgin2 |  |
| Trmt2a |  |  |  | Adgre1 |  |
| Rnf144a |  |  |  | Dennd2d |  |
| Clec4d |  |  |  | Rbfox2 |  |
| Zfp456 |  |  |  | Htr3b |  |
| Ly6g5b |  |  |  | Ajap1 |  |
| Sncaip |  |  |  | Or12d3 |  |
| Ilf3 |  |  |  | Zdhhc16 |  |
| Mcoln2 |  |  |  | Pde1a |  |
| Ankrd10 |  |  |  | Col1a1 |  |
| Mta3 |  |  |  | Zbtb10 |  |
| Zfp444 |  |  |  | Loc101928461 |  |
| Efnb2 |  |  |  | Slc2a14 |  |
| Atl2 |  |  |  | Ttty6b |  |
| Tmem161b |  |  |  | Adora2a-As1 |  |
| Serac1 |  |  |  | Fbxo10 |  |
| Serpinb2 |  |  |  | Gprc5c |  |
| Errfi1 |  |  |  | Yrdc |  |
| Lox |  |  |  | Mettl10 |  |
| Pmaip1 |  |  |  | Srrt |  |
| 9030625A04Rik |  |  |  | Sez6l2 |  |
| Ccl2 |  |  |  | Bend7 |  |
| Stx16 |  |  |  | Ighd |  |
| Bcar1 |  |  |  | Myo3a |  |
| Eftud1 |  |  |  | Sntg2 |  |
| Col12a1 |  |  |  | Nt5e |  |
| Lrrc8b |  |  |  | Dchs2 |  |
| Dbf4 |  |  |  | Loc100996919 |  |
| Asph |  |  |  | Or1d5 |  |
| Eid3 |  |  |  |  |  |
| Ccr9 |  |  |  |  |  |
| Ufd1l |  |  |  |  |  |
| Sqstm1 |  |  |  |  |  |
| Gna13 |  |  |  |  |  |
| Spag9 |  |  |  |  |  |
| Ccdc53 |  |  |  |  |  |
| Rasgrp1 |  |  |  |  |  |
| Col5a2 |  |  |  |  |  |
| Hbegf |  |  |  |  |  |
| Pxmp3 |  |  |  |  |  |
| Gm13476 |  |  |  |  |  |
| Lrrc57 |  |  |  |  |  |
| Cass4 |  |  |  |  |  |
| Tnfsf9 |  |  |  |  |  |
| Ilf3 |  |  |  |  |  |
| Cd44 |  |  |  |  |  |
| Zfp651 |  |  |  |  |  |
| Nlrc4 |  |  |  |  |  |
| Cdkn1a |  |  |  |  |  |
| Rccd1 |  |  |  |  |  |
| A730011L01Rik |  |  |  |  |  |
| Dcun1d2 |  |  |  |  |  |
| Mbtps1 |  |  |  |  |  |
| Odf2 |  |  |  |  |  |
| App |  |  |  |  |  |
| F2r |  |  |  |  |  |
| Btnl9 |  |  |  |  |  |
| Wdr61 |  |  |  |  |  |
| Mpp6 |  |  |  |  |  |
| Gm14023 |  |  |  |  |  |
| Sept9 |  |  |  |  |  |
| Tnfrsf9 |  |  |  |  |  |
| Pqlc1 |  |  |  |  |  |
| Gm17296 |  |  |  |  |  |
| Cacnb3 |  |  |  |  |  |
| Fam160a2 |  |  |  |  |  |
| Dnajc6 |  |  |  |  |  |
| 2700078E11Rik |  |  |  |  |  |
| Osbpl3 |  |  |  |  |  |
| Cisd3 |  |  |  |  |  |
| Hpn |  |  |  |  |  |
| Gpr68 |  |  |  |  |  |
| Marcksl1-ps4 |  |  |  |  |  |
| Cd97 |  |  |  |  |  |
| Rapgef1 |  |  |  |  |  |
| Plek |  |  |  |  |  |
| Cep55 |  |  |  |  |  |
| Fam120b |  |  |  |  |  |
| Thbs1 |  |  |  |  |  |
| Ptges |  |  |  |  |  |
| Iffo1 |  |  |  |  |  |
| 5430405H02Rik |  |  |  |  |  |
| Depdc7 |  |  |  |  |  |
| Fbxw7 |  |  |  |  |  |
| Serpinf1 |  |  |  |  |  |
| Gtpbp5 |  |  |  |  |  |
| Fam73b |  |  |  |  |  |
| Rgs16 |  |  |  |  |  |
| Inpp5a |  |  |  |  |  |
| Rabgef1 |  |  |  |  |  |
| Mcfd2 |  |  |  |  |  |
| Zfp703 |  |  |  |  |  |
| C530028O21Rik |  |  |  |  |  |
| Pdp1 |  |  |  |  |  |
| Hsd11b1 |  |  |  |  |  |
| Aamp |  |  |  |  |  |
| Hmga1 |  |  |  |  |  |
| Ltbp2 |  |  |  |  |  |
| Ssb |  |  |  |  |  |
| Mtmr7 |  |  |  |  |  |
| Hmga1 |  |  |  |  |  |
| Ifi203 |  |  |  |  |  |
| Ust |  |  |  |  |  |
| Pkp4 |  |  |  |  |  |
| Prpsap2 |  |  |  |  |  |
| Raver1-fdx1l |  |  |  |  |  |
| Adam15 |  |  |  |  |  |
| Fstl1 |  |  |  |  |  |
| Cd160 |  |  |  |  |  |
| Ptgfr |  |  |  |  |  |
| Guk1 |  |  |  |  |  |
| Tyk2 |  |  |  |  |  |
| Spc25 |  |  |  |  |  |
| Ttpa |  |  |  |  |  |
| 9430008C03Rik |  |  |  |  |  |
| Tspan2 |  |  |  |  |  |
| Klrk1 |  |  |  |  |  |
| Gas1 |  |  |  |  |  |
| Kifc3 |  |  |  |  |  |
| Zfp691 |  |  |  |  |  |
| 1700109H08Rik |  |  |  |  |  |
| Hmga1 |  |  |  |  |  |
| Cstf3 |  |  |  |  |  |
| Xcl1 |  |  |  |  |  |
| Tgif1 |  |  |  |  |  |
| Nnat |  |  |  |  |  |
| Fance |  |  |  |  |  |
| Zfp819 |  |  |  |  |  |
| Serpinb2 |  |  |  |  |  |
| Slc24a5 |  |  |  |  |  |
| Hdac7 |  |  |  |  |  |
| Pogz |  |  |  |  |  |
| Cd34 |  |  |  |  |  |
| Pop1 |  |  |  |  |  |
| Fiz1 |  |  |  |  |  |
| Aven |  |  |  |  |  |
| Tardbp |  |  |  |  |  |
| Tbx21 |  |  |  |  |  |
| Gpr146 |  |  |  |  |  |
| Pcgf2 |  |  |  |  |  |
| Kitl |  |  |  |  |  |
| Plekhg1 |  |  |  |  |  |
| Cnot4 |  |  |  |  |  |
| Aifm2 |  |  |  |  |  |
| Ppil3 |  |  |  |  |  |
| Dguok |  |  |  |  |  |
| Mcl1 |  |  |  |  |  |
| Lhfp |  |  |  |  |  |
| Tcf3 |  |  |  |  |  |
